# Patterning of Lead Halide Perovskite Device Stacks on CMOS Readout Using Selective Microfabrication Protocols

**DOI:** 10.1002/adma.202523002

**Published:** 2026-03-18

**Authors:** Sergey Tsarev, Erfu Wu, Kyuik Cho, Xuqi Liu, Quang Nhat Dang Lung, Emeric Hartman, Tian Sun, Bekir Turedi, Gebhard J. Matt, Stefanie Frick, Sebastian Siol, Taekwang Jang, Ivan Shorubalko, Sergii Yakunin, Maksym V. Kovalenko

**Affiliations:** ^1^ Department of Chemistry and Applied Biosciences Laboratory of Inorganic Chemistry Zürich Switzerland; ^2^ Laboratory For Thin Films and Photovoltaics Empa – Swiss Federal Laboratories for Materials Science and Technology Dübendorf Switzerland; ^3^ Transport At Nanoscale Interfaces Laboratory Empa – Swiss Federal Laboratories for Materials Science and Technology Dübendorf Switzerland; ^4^ Laboratory of Integrated Systems Department of Information Technology and Electrical Engineering Zürich Switzerland; ^5^ Laboratory For Surface Science and Coating Technologies Empa – Swiss Federal Laboratories for Materials Science and Technology Dübendorf Switzerland

**Keywords:** complementary metal‐oxide semiconductor, image sensor, lead‐halide perovskites, lithography, patterning, monolithic integration, photodetector

## Abstract

Lead halide perovskites represent a promising class of semiconductor materials, notable for their unique optoelectronic properties. However, their application in advanced semiconductor devices, such as CMOS image sensors, photonic integrated circuits, and memristors, requires the development of precise, perovskite‐specific patterning processes compatible with standard cleanroom fabrication. Here, we introduce several key innovations enabling standard microfabrication with lead halide perovskites. First, surface passivation with sorbitan laurate effectively seals the perovskite grain boundaries, enabling the use of standard photoresists (e.g., AZ1518) and aqueous developers on complete device stacks. Furthermore, a modified phosphoric acid etchant, incorporating phenylbutylammonium bromide (PBABr), facilitates the selective etching of transparent conductive oxides (TCOs) such as ITO directly atop the perovskite stack without significant degradation of the active layer. Finally, SF_6_ plasma treatment, using the patterned TCO as a hard mask, selectively converts perovskite in the interpixel gaps into non‐photoactive PbF_x_Br_2‐x_, effectively suppressing lateral cross‐talk. Utilizing this integrated fabrication strategy, we successfully fabricated and characterized a 400 × 400 pixel perovskite CMOS image sensor, where the well‐defined pixels are essential for high spatial resolution and sensor performance. Our results establish a pathway for the development of high‐performance (opto)electronic devices based on lead halide perovskites integrated via standard semiconductor processing methods.

## Graphical abstract

We present a microfabrication and integration strategy for lead halide perovskite photodetectors on electronic readouts. Standard photolithography, aqueous processing, selective transparent electrode etching, and plasma‐assisted pixel isolation enable precise monolithic integration of patterned 400 × 400 perovskite microphotodetector arrays on a CMOS substrate, demonstrating a functional perovskite‐based image sensor.

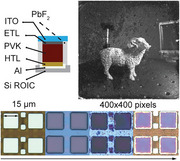

## 1 Introduction

Lead halide perovskites have gained significant attention as exceptional semiconductor materials, particularly in applications such as solar cells [1, 2], light‐emitting diodes (LEDs) [3], and photodetectors [17, 18, 19, 20]. Their remarkable optoelectronic properties, including high absorption coefficients [4], tunable bandgaps [5, 6, 7], and long carrier diffusion lengths [8], make them highly attractive semiconductors for various electronic applications. Nevertheless, integrating perovskites into advanced electronic systems, such as transistor arrays, memristors, photonic integrated circuits, and image sensors, remains challenging [9, 10]. The primary obstacles arise from the material's inherent instability [11, 12] and its incompatibility with conventional semiconductor fabrication environments [13, 14], which typically rely on aqueous lithographic processes and exposure to ambient conditions, both of which rapidly degrade perovskites [15, 16].

The issues described above have been recently tackled by focusing on addressing the challenges of perovskite incompatibility with conventional lithography processes by developing dedicated resist systems [17, 18] that use solvents [15, 19] compatible with perovskites. For instance, PMMA photoresists have been used for e‐beam lithography on perovskites due to their processing with exclusively non‐polar solvents [15, 20, 21]. Alternatively, a double layer of mixed SU‐8 epoxy, or AZ‐5214, AZ MIR 701, and PMMA, can be used for patterning perovskite films via top‐down lithography [22, 23, 24]. Lift‐off methods have also been widely explored for patterning perovskite films [25, 26, 27]. Furthermore, various unconventional methods, such as microfracture patterning [28], crack propagation [29], liquefaction of perovskite powders [30], or hydrophobic‐hydrophilic surface coating, have been utilized to form perovskite micropatterns [31]. Despite this significant progress, most reported perovskite patterning strategies remain incompatible with standard cleanroom photolithography workflows. Solution‐processed techniques based on surface energy modification and microfracture often struggle to achieve high fill factors and reproducible yields, and are typically restricted to patterning the active layer rather than whole device stacks. E‐beam lithography is limited by small pattern areas and low throughput, while epoxy‐based photoresists are generally intended for permanent structures, making subsequent removal difficult [32]. Orthogonal fluorinated solvent systems often have prohibitive costs for mass production scales and are difficult to access [19]. In approaches relying on polymer or polymer–resist composite protection layers, applied before coating standard resists, an additional step may be required to fully remove the protective coating after the lithography step, depending on the specific process flow, which can increase process complexity. In contrast, the backbone of modern semiconductor manufacturing relies almost exclusively on positive‐tone Novolak‐based photoresists and aqueous alkaline developers, which account for the vast majority of optical lithography processes in industrial CMOS fabrication. However, direct application of these resists to perovskite device stacks has remained largely inaccessible due to rapid degradation in water‐based environments, forcing the use of protective polymer interlayers or unconventional patterning routes.

In this work, we demonstrate a fully conventional top‐down photolithography process using standard positive Novolak photoresists and aqueous developers directly on a functional lead‐halide perovskite device stack, without intermediate encapsulation layers and glovebox processing. Our approach introduces several key innovations to ensure compatibility with standard cleanroom CMOS fabrication processes. These include: (1) thermal evaporation of perovskites for reproducible and conformal deposition, even on chip surfaces with complex topography; (2) application of an organic nonionic surfactant as a protective passivation layer to enable water‐based processing; (3) controllable wet etching of transparent conductive electrode layers atop perovskite films; and (4) conversion of interpixel perovskite regions into insulating lead fluoride glass to minimize electrical crosstalk and enhance device stability. This combined approach enables the fabrication of fine structures with high reproducibility. Importantly, the novelty of our approach does not lie in introducing a new resist chemistry, but in establishing a process‐compatible materials and etching toolbox that enables perovskite integration within existing CMOS microfabrication infrastructure. To demonstrate its capabilities, we fabricated a 400 × 400‐pixel patterned perovskite CMOS image sensor using standard wet‐lithography techniques. Compared to a non‐patterned reference device, the fabricated sensors demonstrated significant improvement in the modulation transfer function, highlighting the importance of optical and electrical separation of pixels for the development of micro‐scale perovskite photodetectors. In general, this approach allows fabrication of the next generation of perovskite electronic devices, directly integratable on silicon at a large scale with high precision.

## 2 Results and Discussion

### 2.1 Enabling Aqueous Processing for Lithography on Perovskite Device Stacks

**FIGURE 1:**
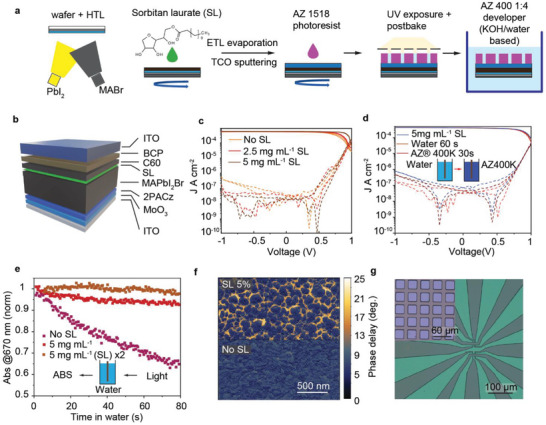
Perovskite photodetector stack fabrication and patterning. (a) Schematic representation of the layers deposition and patterning process. (b) Complete device structure used in this study. (c) Current density‐voltage (*J‐V*) characteristics of photodetectors measured in the dark and under 1 mW cm^−2^ LED irradiation (626 nm), as a function of sorbitan laurate (SL) concentration in the passivating solution. (d) *J–V* curve of an SL‐passivated (5 mg mL^−1^) perovskite photodetector before and after sequential immersion in water (60 s) and AZ 400K developer (30 s). (e) Change in absorbance of the perovskite device stack over time during immersion in an aqueous solution, comparing non‐passivated and SL‐passivated stacks. For SLx2 sample, 5 mg mL^−1^ SL was spincoated on top of the completed device. (f) AFM phase image of pristine and SL‐passivated perovskite films. (g) Examples of photoresist patterns obtained on a perovskite device stack.

An integration of lead halide perovskite films with conventional cleanroom processing is challenging due to their sensitivity to water, air, and polar solvents commonly used in standard fabrication. Most standard lithography processes rely on Novolac‐based resins for UV and chemically amplified resists for DUV patterning, both typically developed using aqueous alkaline solutions. In this work, we enable the processing of perovskites in ambient air and water‐based environments by applying sorbitan laurate (SL) passivation, which significantly enhances the stability of perovskite device stacks during water immersion. This passivation technique, previously reported to improve moisture stability in perovskite solar cells [33], is adapted here to facilitate lithographic patterning on perovskite devices (Figure 1a). A standard *p‐i‐n* stack [34] with a 2‐(9H‐carbazol‐9‐yl)ethylphosphonic acid HTL (2PACz), the perovskite layer, C60/BCP (Bathocuproine) electron‐selective layers, and ITO (Indium Tin Oxide) or Ag electrodes is sequentially deposited on glass/ITO substrates using physical vapor deposition (Figure 1b). After the deposition of perovskite, the films are passivated by spin‐coating a 5 mg mL^−1^ solution of SL in isopropanol.

The SL‐passivated devices exhibited a significant reduction in dark current density and an increase in open‐circuit voltage (*V*_OC_), suggesting a decrease in perovskite surface trap density [35] (Figure 1c). Statistical analysis of device performance as a function of SL concentration is provided in Figure S1. Remarkably, the complete device stacks displayed outstanding stability when immersed sequentially in water (60 s) and aqueous AZ 400K resist developer (30 s), as confirmed by comparing detector performance before and after immersion (Figure 1d). More detailed dynamics of degradation of device stacks during water immersion were monitored by changes in optical absorption at the wavelength of 670 nm (Figure 1e). The absorbance of the reference stack (without SL passivation) decreased rapidly, whereas SL‐passivated stacks showed minimal degradation over short timescales. An additional layer of SL coated on the ITO electrode using the same deposition protocol further improved the water immersion stability of the devices.

Contrary to some implications in earlier work [33], we did not observe significantly enhanced water stability of the perovskite film modified by SL without the top electrode. Therefore, we attribute the improved water stability of the device stack primarily to the SL layer improving barrier properties of the ITO electrode, particularly near grain boundaries. To test this hypothesis, we compared phase‐contrast atomic force microscopy (AFM) images of pristine and SL‐treated films to investigate morphological changes in the films. Areas with delayed phase reveal that SL preferentially accumulates at grain boundaries, effectively sealing the perovskite film surface (Figure 1f), which likely contributed to the growth of ETL/ITO layers with improved barrier properties. Longer‐term immersion tests of the full device stacks (Figure S2) revealed degradation after approximately 6 min, likely initiated from the device edges (by edge delamination). However, this processing window is sufficient for typical photoresist development and rinsing steps.

These findings enabled us to perform standard photolithography using positive DNQ‐Novolak resists, such as AZ1505 and AZ1518. We successfully obtained features down to a 1 µm resolution, limited by the optical capabilities of our lithography equipment. Examples of patterned structures are presented in Figure 1g.

### 2.2 Selective Patterning of Top Electrode Layers

**FIGURE 2:**
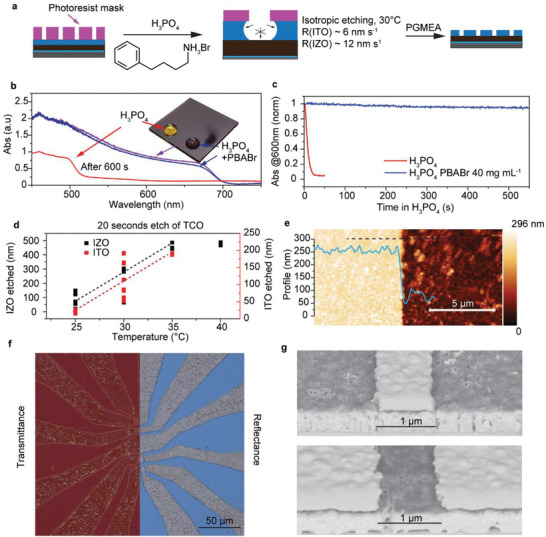
Patterning of transparent electrode layers in perovskite device stacks. (a) Schematic representation of the TCO patterning process. Changes in absorption spectra (b) and corresponding dynamics of optical absorbance at 600 nm (c) of MAPbI_2_Br perovskite film, without any additional treatments, immersed in H_3_PO_4_ and H_3_PO_4_:PBABr. Each measurement was performed on n = 3 independent samples. (e) AFM topography map and a profile of the etched ITO pattern on the perovskite device stack. (f) Optical micrographs (left in transmittance, right in reflectance modes) showing a test pattern of ITO etched on a device stack. (g) Scanning electron microscopy scan showing high‐resolution (1 µm) ITO structures: line (top) and gap (bottom) patterns achieved by developed H_3_PO_4_:PBABr etching.

To achieve a reproducible pixelization process across the entire device area, selective and controllable etching processes are required. Here, we demonstrate the wet etching of transparent conductive oxides (TCOs) directly on lead halide perovskite layers (Figure 2a). To mitigate perovskite instability in aqueous acids, we selected concentrated phosphoric acid (H_3_PO_4_, 85%) as the base etchant, leveraging the low solubility of lead phosphate (Pb_3_(PO_4_)_2_) in water (0.1 µm at pH 6) [36], which should limit the dissolution of the perovskite's lead component. Preliminary experiments confirmed that perovskite films degraded significantly slower in phosphoric acid compared to other common aqueous acid solutions, such as sulfuric, hydrochloric, and citric acids. Despite the reduced dissolution rate, chemical degradation of the perovskite films was still observed in 85% H_3_PO_4_, which we attribute to the solubility of its organic components (e.g., methylammonium) in the aqueous environment. To inhibit this degradation, we introduced phenylbutylammonium bromide (PBABr) into the phosphoric acid solution, aiming to suppress the dissolution of the organic phase. At a concentration of 20 mg mL^−1^ PBABr in H_3_PO_4_, we observed negligible changes in the optical absorbance of perovskite thin film at 600 nm over 10 min (Figure 2b,c). This suggests that PBABr acts as an effective stabilizing agent, potentially by forming a protective layer or passivating the perovskite surface, thereby hindering interaction with the aqueous medium. Notably, the chemically modified phosphoric‐acid etchant introduced here enables, for the first time, selective wet etching of TCO electrodes directly on perovskite device stacks that are otherwise incompatible with pristine phosphoric‐acid chemistry.

The etch rates for ITO and indium zinc oxide (IZO) thin film electrodes in the H_3_PO_4_/PBABr solution were systematically evaluated as a function of temperature. At 30 °C, the mean etch rate for IZO was approximately 12 ± 7 nm s^−1^, while ITO etched slower at 6 ± 4 nm s^−1^ (Figure 2d). Due to the significant substrate‐to‐substrate etch rate variation observed at 30 °C, we conducted further processing at 35 °C (mild over‐etching conditions) to improve consistency. It should be noted that these dissolution rates apply only to low‐temperature, in‐house sputtered ITO films, while high‐density commercial crystalline ITO, deposited at elevated temperatures, exhibited negligible dissolution under identical conditions.

ITO patterning was performed using an AZ1518 photoresist mask, defined using the previously described lithography process. As it was previously tested (Figure 2b,c), the H_3_PO_4_/PBABr etchant demonstrated high selectivity, effectively etching the TCO layer with minimal chemical attack on the underlying perovskite, allowing the precise fabrication of ITO top electrode patterns directly on the device stack. The resulting patterns exhibited sharp, well‐defined edges, as shown in the AFM topography scan (Figure 2e). Optical micrographs confirmed clean etching in the exposed regions, while the photoresist‐masked areas remained intact (Figure 2f; Figure S3). Additionally, PL spectra indicate only minor changes after ITO etching, suggesting that the bulk optoelectronic properties of the perovskite films remain preserved (Figure S4).

We then assessed the minimum feature size and gap achievable with this etching process. Using AZ1505 resist and an optimized lithography recipe, we etched 200 µm‐long ITO lines with varying widths and spacings in perovskite device stacks deposited on a glass substrate. The optimized results, presented in Figure 2g, demonstrate the successful formation of 1 µm‐wide ITO lines and 1 µm‐wide gaps, dimensions compatible with pixel sizes required for high‐resolution image sensors.

### 2.3 Patterning of Perovskite Pixels

Following the successful patterning of TCO layers, we developed an approach for the lateral isolation of pixels from neighboring ones by conversion of the perovskite semiconductor into a non‐optically active material using a plasma‐enhanced gaseous halide‐exchange process, further named as “conversion.” As will be discussed later, we used this process on the image sensors with a monolithic perovskite layer, where patterning of the perovskite stack is crucial to minimize electrical cross‐talk between adjacent pixels. This can be achieved either by physically removing the perovskite between pixels [27] or by converting it into a non‐optically active material. In this study, we employed a previously reported method involving gaseous plasma‐assisted halide exchange used to convert MAPbI_3_ perovskite into lead fluoride glass [19].

**FIGURE 3:**
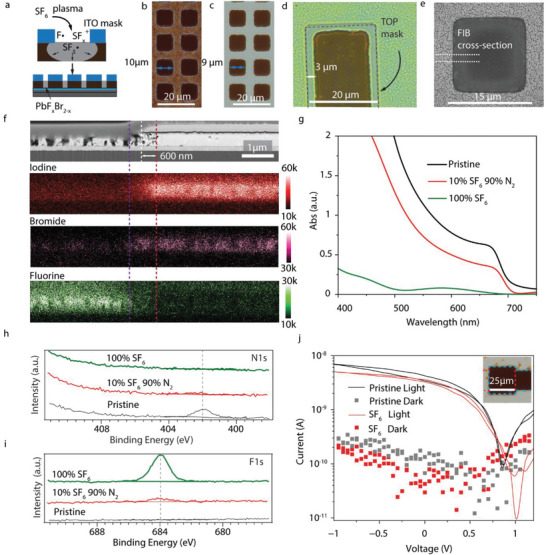
Patterning of perovskite pixels via SF_6_ plasma conversion. (a) Schematic illustration of the perovskite‐to‐PbF_x_Br_2‐x_ conversion process in SF_6_ plasma using a top‐patterned ITO as a mask. (b,c) Optical micrographs of a pattern with 10 µm perovskite squares during plasma conversion with diluted SF_6_, showing partially converted (b, 20% SF_6_ 80% N_2_ 100 W, 50 s) and fully converted (c, 20% SF_6_ 80% N_2_ 100 W, 110 s) perovskite. We observed minimal lateral over‐conversion of approximately 0.5 µm on each side. (d) Optical micrograph of a 20 µm stripe in a perovskite device stack, treated with pure SF_6_ plasma (200 W, 160 s), showing a maximum lateral over‐conversion of ∼3 µm underneath the ITO edge. (e) Scanning electron microscopy (SEM) image of a 15 µm pixel after diluted SF_6_ plasma conversion (20% SF_6_ 80% N_2_ 110 s), acquired using a backscattered electron detector. The focused ion beam (FIB) cross‐section location is indicated by the white dashed line. (f) FIB cross‐sectional image of the pixel edge shown in (e), together with energy‐dispersive X‐ray (EDX) elemental maps of iodine, bromine, and fluorine. (g) Optical absorption spectra of a bare perovskite film after exposure to diluted SF_6_ plasma (10% SF_6_ 90% N_2_, 100 W, 10 s) or SF_6_ plasma at (200 W, 20 s). (h,i) X‐ray photoelectron spectroscopy (XPS) detail spectra of the N1s (h), F1s (i) regions. Data indicated the removal of methylammonium species from the surface after diluted SF_6_ plasma exposure and subsequent progressive fluorination. (j) Current density‐voltage characteristics of a lithographically patterned (H_3_PO_4_/PBABr etch + 100% SF_6_ 60 s, 200 W) 25 × 25 µm^2^ perovskite photodetector before and after SF_6_ plasma treatment. Inset (j): Optical microphotograph of the corresponding device.

The procedure is schematically illustrated in Figure 3a. Briefly, perovskite films with pre‐patterned ITO electrodes serving as masks were exposed to sulfur hexafluoride (SF_6_) plasma in a reactive ion etching (RIE) chamber. Fluorine radicals generated in the plasma rapidly substitute halogen ions in the perovskite lattice. Since organic components and common electron transport layers (like BCP/C60) are etched by fluorine‐containing plasma, this process simultaneously removes these layers in the exposed regions, eliminating the need for separate removal steps.

To probe the lateral resolution limits of the patterning process, we performed a controlled perovskite conversion process using diluted SF_6_ plasma. By varying the plasma exposure time, we observed a gradual transformation of the films, further referred to as “partially converted” (Figure 3b) and “fully converted” (Figure 3c) states. Partially converted samples preserved the nominal 10 µm lateral dimensions, whereas fully converted samples exhibited a reduction of the patterned feature size from 10 to approximately 9 µm, which is expected for an isotropic propagation of the plasma‐induced conversion front. Under aggressive overconversion conditions using pure SF_6_ plasma for 160 s, the maximum lateral overconversion was approximately 3 µm (Figure 3d), which was likely limited by the diffusion length of fluorine‐containing radicals within the perovskite film.

To gain insight into the chemical and structural evolution induced by the plasma treatment, we performed focused ion beam (FIB) cross‐sectional analysis on fully converted samples. A top‐down SEM image acquired using a backscattered electron detector before the FIB cut is shown in Figure 3e. The uniform contrast across the pixel area suggested a homogeneous distribution of heavy elements within the masked perovskite region. The FIB cross‐section (Figure 3f) reveals the formation of a porous structure in the plasma‐exposed regions, as well as a ∼600 nm laterally converted zone underneath the ITO mask edge, in good agreement with optical microscopy observations. Energy‐dispersive X‐ray (EDX) elemental mapping shows that fluorine is confined to the plasma‐exposed regions, while the over‐converted zone beneath the ITO mask exhibits an increased bromine concentration accompanied by a reduced iodine content.

Importantly, the partially converted samples did not exhibit a significant wavelength shift of the absorption spectrum, which would be indicative of iodide‐to‐bromide exchange in the bulk of the perovskite film (Figure 3g). To resolve local compositional variations that are not captured in area‐averaged spectra, we performed spatially resolved absorption mapping. These measurements reveal a region with a broad absorption feature, localized at the pixel edge, extending over a lateral length scale of approximately 0.5 µm (Figure S5). We attribute this spectrum to the formation of a variety of perovskite‐like domains, with different bromide to iodide ratios.

X‐ray diffraction (XRD) analysis (Figure S6) reveals a gradual decrease in MAPbI_2_Br reflection intensity and the formation of PbI_2_ as an intermediate phase in the partially converted samples. This suggests that the organic component of the perovskite (methylammonium) is preferentially removed at early stages of plasma treatment, resulting in the formation of lead‐based salts that are optically inactive in the perovskite absorption region. After exposure to pure SF_6_ plasma, only weak residual perovskite peaks remain, indicating the formation of an amorphous, likely mixed lead fluoride and lead halide species with residual perovskite domains.

X‐ray photoelectron spectroscopy (XPS) was used to further investigate the surface chemistry of partially and fully converted films. The N 1s and F 1s core‐level spectra (Figure 3h,i) show that ammonium‐containing species are removed from the surface already at the partially converted stage, prior to significant fluorine incorporation. This observation is consistent with the preferential loss of the organic cation and the formation of lead‐halide‐rich surface species, in agreement with XRD results. In the fully converted samples, fluorine becomes the dominant anion at the surface, while a small amount of bromine remains detectable, yielding an approximate Br: F ratio of 1:5 (Table S2). More details of Pb 4f, I 3d, Br 3d, and C 1s core levels, as well as quantitative surface compositions, are provided in Figures S7 and S8, and Table S2. Taken together, the XPS, XRD, and optical data support the following conversion mechanism: initial plasma exposure removes the organic component of the perovskite and induces partial halide reorganization with limited fluorine incorporation; prolonged exposure results in extensive halide exchange and collapse of the perovskite lattice, yielding an amorphous lead fluoride–bromide material.

To evaluate the electrical properties of the converted and partially converted materials, we fabricated photoconductors by depositing perovskite films onto interdigitated electrode arrays comprising 400 × 400 electrode pairs with a 10 µm channel length per device. Reference perovskite films exhibited pronounced hysteresis, light‐induced switching behavior, and electrical breakdown at biases exceeding ±1 V (Figure S9), consistent with trap‐rich, unpassivated methylammonium‐based perovskite films. In contrast, partially converted films showed a dramatic reduction in hysteresis, increased breakdown voltage, and significantly reduced dark current density. Given the XPS evidence for limited fluorine incorporation in these samples, we attribute this improvement to plasma‐induced passivation of surface halide vacancies. Fully converted films displayed no measurable photoresponse and a modest increase in dark current relative to pristine and partially converted films, consistent with the presence of electronic trap states in the optically inactive converted material.

Overall, the conversion process results in the formation of a non‐photoconductive material between pixels, while simultaneously producing passivated perovskite edges. This is particularly advantageous for microscale photodetector fabrication, as it avoids the edge damage and leakage currents commonly associated with physical etching processes and enables the realization of small‐area detectors where edge effects would otherwise dominate.

To assess the impact of the TCO lithography and etching sequence on photodiode performance before perovskite conversion, we fabricated large‐area (4 × 4 mm^2^) detectors. The top ITO electrode was patterned using our photolithography/wet‐etching protocol, or for control devices, by deposition through a shadow mask. After initial electrical characterization, all devices were subjected to the SF_6_ plasma (conversion) followed by oxygen plasma cleaning (required for image sensor fabrication steps, such as surface cleaning and pad opening). At every intermediate stage (i.e., H_3_PO_4_ etch, SF_6_ conversion, resist‐residue removal) and in the final state, we found no performance loss attributable to the lithography route. On the contrary, devices patterned by lithography showed a slightly higher *V*_OC_, which we attributed to the absence of mechanical damage associated with shadow mask alignment and sputtering damage accumulated at pattern edges. Otherwise, the IV performance was comparable between the two patterning methods (Figure S10). Both patterned and non‐patterned detectors show an extended linear (α = 1) dynamic range of 103 dB, limited by maximum accessible optical power (Figure S11) and detectivities of 2.8∙10^11^ Jones and 2.2∙10^11^ Jones for patterned and non‐patterned detectors accordingly. Additionally, we summarize the photodetector performance and provide a comparison with representative state‐of‐the‐art devices in Table S3.

Encouraged by these results, we fabricated microphotodetectors using the full patterning sequence, including perovskite SF_6_ conversion. Figure 3d shows an optical micrograph of a 25 × 25 µm^2^ detector fabricated on a glass substrate, demonstrating clean etching and clearly defined pixel edges achieved with the optimized process. To showcase scalability for imaging applications, we also fabricated arrays of perovskite pixels on a glass substrate with feature sizes down to 4 and 15 µm (Figure 3e), dimensions suitable for CMOS sensors. Electrical characterization of these microphotodetectors confirmed performance comparable to 4 × 4 mm^2^ detectors, yielding a nominal external quantum efficiency (EQE) of 86% at 626 nm calculated from photocurrents at 0 V (Figure 3f) and dark currents at the equipment noise floor. Because the precise active area of such small pixels is difficult to define, this EQE value is likely overestimated; using the 67% EQE measured for large‐area detectors (Figure S12) as a reference, the true EQE of the micro‐devices is likely ≤67%. Additionally, the detectors exhibited fast temporal response, with a measured time constant of 110 ns under pulsed 488 nm laser excitation (Figure S13).

### 2.4 Perovskite Photodetector Integration Onto a CMOS Readout Circuit Microchip

**FIGURE 4:**
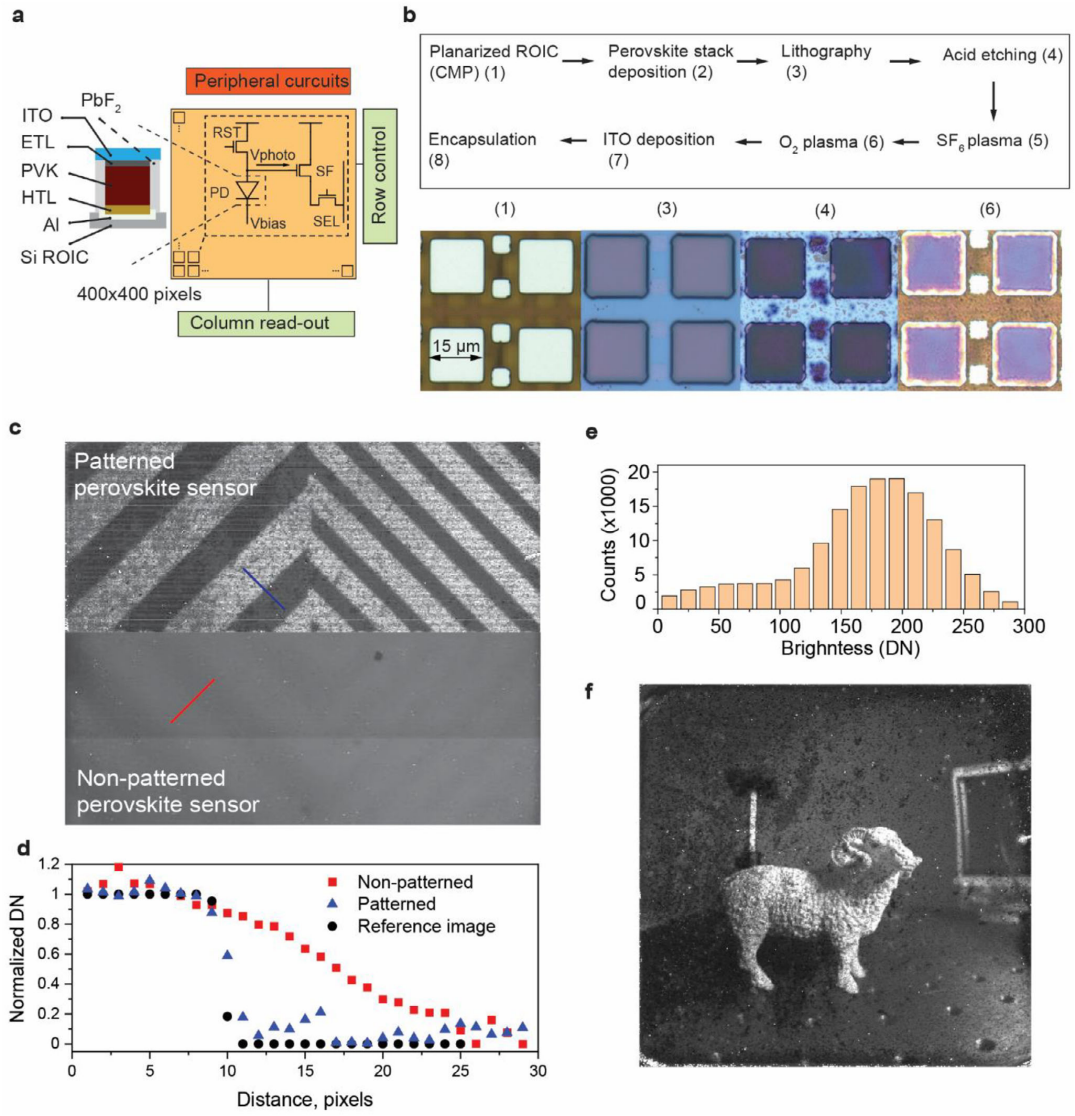
Integration of perovskite photodetectors on Si CMOS readout. (a) Layout of the 400 × 400 pixel perovskite image sensor. The sensor employs a three‐transistor (3T) pixel configuration, with the source‐follower gate connected to the p‐collector of the perovskite photodiode via an aluminum electrode. (b) Schematic of the perovskite sensor fabrication process. Lower panels show optical micrographs (all in reflection mode) of steps (1) planarized ROIC aluminum electrodes, (3) SU‐8 photoresist pattern on perovskite, (4) H_3_PO_4_‐etched ITO patterns, and (6) SF_6_‐plasma‐processed pixels after resist removal. (c) Raw sensor photos of a diagonal‐line test pattern acquired with patterned versus non‐patterned perovskite. (d) Normalized, background‐subtracted edge profiles compared with the original emitted pattern. (e) Histogram of pixel photoresponses, in DN (Digital Numbers), obtained from a gray image at 200 nW cm^−2^ uniform 460 nm LED light. Histogram obtained from 400 × 400 pixels. Pixel statistics were calculated from a single sensor array measurement. (f) Raw photo of a toy lamb positioned on an optical table, taken with the patterned perovskite CMOS sensor.

We first validated process scalability on glass, patterning 400 × 400 arrays of 15 × 15 µm pixels with the protocol defined above. Pixel uniformity was confirmed by optical inspection (Figure S14a). When the same process was transferred to chemically‐mechanically polished (CMP) custom‐designed CMOS readout integrated circuit (ROIC) dies, partial pixel degradation and water ingress into the films were observed (Figure S14b), attributable to an incomplete planarization procedure and the presence of polishing slurry residuals coming from a shared research‐grade polishing equipment. Taking into account imperfect planarization and dust particles coming from outside the cleanroom processes, to maximize pixel yield, we introduced several chip‐specific fabrication protocol modifications, such as replacing all spincoating steps for PVD‐depositions, minimizing the number of inter‐tool transfers, and replacing the photoresist from AZ1518 to SU‐8 2001 (non‐aqueous photoresist). The complete *p‐i‐n* perovskite stack was then deposited on the ROIC chips (Figure 4a) through a window shadow mask that defined the 400 × 400‐pixel imaging area. Using an optimized fabrication protocol, no solvent‐related degradation was visually observed (Figure S14c). We calculated 93% pixel yield in a batch of 24 devices (Figure S15). The full fabrication sequence, along with micrographs after key steps 1, 3, 4, and 6, is summarized in Figure 4b.

To quantify the benefits of perovskite patterning, we first imaged a high contrast diagonal bars test pattern with sensors employing patterned and unpatterned perovskite layers. The patterned sensor resolved edges within 2 pixels, comparable with the sharpness of the emitted test image (Figure 4c, top). In contrast, the unpatterned device exhibited severe blooming consistent with long lateral carrier diffusion lengths of perovskite thin films, yielding an edge spread width of ∼15 pixels (Figure 4c, bottom). The edge profiles across a projected diagonal line are presented in Figure 4d. These results underscore that device stack level patterning is essential for thin film photodiodes possessing high carrier mobility.

We observed that the produced CMOS sensor array showed significant pixel‐to‐pixel variability, which we attributed to chip planarization non‐uniformities and process variability. We classify a pixel as active when its illuminated output, after dark‐frame subtraction, lies between ±50% of the median signal value. According to this criterion, 86% of the pixels are active (Figure 4e). Within this subset, the photo‐response non‐uniformity of ≈37% r.m.s (σ/µ) was calculated, which is high compared with consumer‐grade CMOS imagers but acceptable for a proof‐of‐concept device. Linearity, sensitivity, and dynamic range were assessed by measuring the pixel output as a function of incident irradiance (Figure S16). The array detects light power as low as 40 nW per pixel at an SNR equal to 2 and provides an array‐averaged linear dynamic range of ≈40 dB (two decades).

Finally, we demonstrate the imaging capability of the sensor by collecting a photo from a scene (Figure 4f). The sensor demonstrated acceptable raw image quality, highlighting the suitability of perovskite as an imaging material compatible with integration into conventional silicon microcircuits.

## 3 Conclusion

We present a complete CMOS‐compatible technological protocol for patterning of lead halide perovskite device stacks and their monolithic integration with modern electronic platforms, based on several novel approaches. First, a sorbitan‐laurate surface treatment makes the perovskite device stack compatible with aqueous photoresist developers. Second, a modified phosphoric‐acid etchant selectively removes transparent conductive oxides (TCOs) deposited on the perovskite without damaging the underlying film. Third, gaseous halide exchange in an SF_6_ plasma isolates adjacent pixels at the micrometre scale. Using this process toolbox, we directly integrated patterned perovskite photodiodes onto a CMOS imager, achieving substantially lower optical crosstalk than the unpatterned control sample. The methods introduced here are broadly applicable and could accelerate the incorporation of perovskites into next‐generation optoelectronic devices, including, but not limited to, image sensors, micro‐LEDs, memristors, and microscale photodetectors.

## 4 Experimental Section

All chemicals were used as supplied, without further purification.

### 4.1 Perovskite Precursors

Methylammonium bromide (99.99%, Great Cell Solar), lead iodide (Thermo Scientific, 99%), Sorbitan laurate (99%, Thermo Scientific).

### 4.2 Sputtering Targets

Targets are obtained from Angstrom Engineering: Indium Zinc Oxide (IZO) (In_2_O_3_+ZnO 90:10 wt.%) Sputtering Target—Ø3“, Purity: 99.9%, Thickness: 1/8”, Bonded to 1/8″ OFHC backing plate. Indium Tin Oxide (ITO) (90:10 wt.% In_2_O_3_:SnO_2_) Hollow Cathode Sputtering Target—Ø147 mm × 52 mm, Purity: 99.99% Indium bonded to OFHC backing plate.

### 4.3 Solvents

Anhydrous (AcroSeal) Ethanol and Isopropanol were purchased from Acros (99%) and used inside a nitrogen‐filled glovebox.

### 4.4 Charge‐Transport Materials and Electrodes

Molybdenum Trioxide (99.99%, Alpha‐Aesar), 2PACz or (2‐(9H‐carbazol‐9‐yl)ethyl)phosphonic acid; (>99%, Lumtec), C60 (99.9% SES Research), Bathocuproine or 2,9‐Dimethyl‐4,7‐diphenyl‐1,10‐phenanthroline (99%, Ossilla).

### 4.5 Sensor Fabrication

The glass/ITO substrates (16 Ohm sq^−1^, Kintec) were subjected to a sequential cleaning process using 2% Hellmanex solution, water, acetone, and isopropanol, after which they were treated with UV ozone for 10 min. Following this, thermal evaporation was employed to deposit 30 nm of molybdenum oxide. Subsequently, the hole transport layer (HTL) precursor, 2PACz (1.2 mg mL^−1^ in ethanol), was spin‐coated at 3000 rpm for 30 s. The samples were transferred to a vacuum chamber where methylammonium bromide (MABr) and lead iodide (PbI_2_) were co‐evaporated onto the substrate held at 20 °C. Deposition of perovskite was controlled by PbI_2_ rate (0.48 Å s^−1^) in order to reach an effective thickness of 300 nm of PbI_2_, corresponding to a total perovskite thickness of 450 nm. MABr partial pressure was kept constant via a fixed crucible temperature (132 °C), previously calibrated for stoichiometry. After the deposition, a passivation layer of sorbitan laurate (SL) was applied by spin‐coating a 5 mg mL^−1^ solution in isopropanol at 3000 rpm, followed by annealing at 80 °C for 2 min on a hotplate. The electron transport layer (ETL) stack, consisting of C_60_ (40 nm) and bathocuproine (BCP, 8 nm), was then deposited via thermal evaporation at rates of 0.2 and 0.15 Å s^−1^, respectively. Device stacks were completed by sputtering the 180 nm ITO transparent top electrode. Finally, a second SL passivation layer was applied using the same spin‐coating and annealing procedure as the first. For photoconductor devices, perovskite was directly deposited on cleaned glass/interdigitated ITO substrates. Each photoconductor consisted of 400 pairs of interdigitated ITO electrodes with dimensions of 190 × 10 µm2 electrodes and 10 µm channel spacing. After perovskite deposition, the films were exposed either to diluted SF_6_/N_2_ plasma (10% SF_6_ 90% N_2_,100 W 10 s) or to pure SF_6_ plasma (200 W 20 s), corresponding to partially and fully converted samples accordingly.

### 4.6 Patterning

For top‐down lithography on the device stacks, AZ1518 positive photoresist was spin‐coated at 4000 rpm (yielding ∼1.8 µm thickness) and annealed at 110 °C for 120 s. Patterns were defined by exposing the resist to 150 mJ cm^−2^ of 436 nm light (g‐line) from a mercury lamp of a mask aligner. The exposed resist was developed for 30 s in AZ 400K developer diluted 1:4 with DI water, followed by a 30‐s rinse with DI water and annealing at 110 °C for 120 s. For defining minimal features (∼1 µm), AZ1505 photoresist was used with similar processing steps. Any residual resist in developed areas was removed using an oxygen plasma etch (100 W, 75 mTorr O_2_, 20 s). The patterned photoresist served as a mask for wet etching the TCO layer. Etching was performed by immersing the sample in 85 % H_3_PO_4_ containing 20 mg mL^−1^ phenylbutylammonium bromide (PBABr) maintained at 35 °C for an optimized time (1–1.5 min, depending on the substrate, to guarantee full TCO removal). After etching, the phosphoric acid residue was washed using hexanol two times, followed by an isopropanol wash. The photoresist residue was washed using propylene glycol methyl ether acetate. The underlying layers in the exposed regions were converted to PbF_x_Br_2‐x_, (perovskite) or etched (C_60_/BCP) in SF_6_ plasma (200 W, 75 mTorr SF_6_, 40 s) in a reactive ion etching (RIE‐80, Oxford Instruments) system.

### 4.7 Image Sensor Preparation

Readout integrated circuits (ROICs), custom‐designed and fabricated using a commercial 180 nm CMOS process, served as the starting substrates. ROIC chips underwent chemical‐mechanical polishing (CMP) for planarization and pixel pad opening. The p‐i‐n perovskite device stack was deposited onto the prepared ROIC pixel array area using physical vapor deposition through shadow masks. Before the deposition, bonding pads were protected using a 1 µm SU‐8 mask. MoO_3_ (30 nm) and 2PACz (4 nm) were thermally evaporated through a 10 mm square window mask, defining the sensor active area. After deposition, substrates were rinsed for 30 s in isopropanol in a cleanroom environment. Perovskite was deposited using 0.48 and 0.55 Å s^−1^ for the first 1500 Å and the second 1500 Å measured as PbI_2_ deposition rate. MABr rate was identical to that in the fabrication protocol above. 40 nm C_60_, 8 nm of BCP, and 2 nm Mg layers were deposited at 0.2, 0.15, and 0.1 Å s^−1^, respectively. After a shadow mask change to an 11 mm square mask, ITO was sputtered using an optimized 3‐stage RF sputtering process (in 30 mTorr Ar, at 30 W for 5 min, in 10 mTorr Ar at 30 W, for the first 50 nm, then 2 mTorr Ar‐O_2_ (0.02%) at 90 W for the next 130 nm). SU‐8 2002 was coated on the ROIC and patterned using standard photolithography methods, following ITO etching in H_3_PO_4_ with 20 mg mL^−1^ PBABr for 90 s, washing, and SF_6_ plasma conversion for 60 s. The sensors’ active area was encapsulated with SU‐8 2002 resist, and the sensors were wirebonded to chip carriers using 8 µm Al wires.

### 4.8 External Quantum Efficiency

The External Quantum Efficiency (EQE) spectra were measured in the wavelength range of 300 to 800 nm, utilizing a QE system (Model QE‐R from Enli Tech). The measurements were conducted under near‐dark test conditions with a chopper frequency set at 210 Hz.

### 4.9 Optical Measurements

Absorption spectra in the UV–vis range for perovskite thin films were obtained in transmission mode using a Jasco V‐670 spectrometer. Fluorolog iHR 320 Horiba Jobin Yvon spectrofluorimeter equipped with a PMT detector was used to acquire steady‐ state PL spectra from films. For optical absorption mapping, the spectra were acquired using an Olympus IX 81 inverted microscope equipped with a tungsten‐halogen lamp. One of the optical output ports was coupled to an Andor Shamrock 301i monochromator fitted with an iDus DV420A camera. The microscope was operated with a 60× objective (Olympus LUCPlanFLN, NA 0.7) with the cover glass correction set to 1 mm. The camera has a resolution of 1024 × 254 pixels. With the monochromator entrance slit fully opened to 2.5 mm, the field of view is 104 × 32 µm^2^. Spectra were recorded using a 100 µm slit width, corresponding to a spatial resolution of approximately ∼1.3 µm in the direction perpendicular to the slit. The 254 spectra collected along the slit (covering 104 µm) provide a spatial sampling of ∼0.41 µm.

### 4.10 Transient Photocurrent Measurements

Transient photocurrents were measured as the voltage drop across a 470 Ohm load resistor. The voltage signal was amplified by 40 dB using a low‐noise broadband amplifier (Femto HVA‐200M‐40‐F) and recorded with a digital oscilloscope (Tektronix MSO44). Optical excitation was provided by a solid‐state ps laser (BDL‐488‐SMN, Becker & Hickl GmbH) emitting at 488 nm, with pulse durations in the range of 40–90 ps and a pulse energy of 12 pJ.

### 4.11 X‐Ray Photoelectron Spectroscopy Measurements

X‐ray photoelectron spectroscopy (XPS) measurements were performed on a PHI Quantera employing monochromatized Al Kα radiation with a beam power of 7 W and a voltage of 15 kV. Three measurement areas of each 500 × 1000 µm^2^ were scanned per sample to enable sample statistics and to avoid the formation of metallic lead species during the course of the measurement, which was controlled by recording short Pb 4f spectra before and after the detailed measurements of the Pb 4f, C 1s, N1s, I 3d, Br 3d, F 1s, O 1s regions. The measurements were performed at pressures <5∙10^−9^ Torr using charge compensation by an electron neutralizer. The thin film samples deposited on glass were taped onto a metal plate, which was screwed onto the equipment's sample holder, leading to a floating configuration to avoid differential charging issues [37]. The binding energy was referenced to the aliphatic C 1s peak to 284.8 eV [37]. The compositional ratios X/Pb were determined after Shirley background subtraction and employing the respective relative sensitivity factors of the equipment manufacturer.

### 4.12 Cross‐Section Scanning Electron Microscopy

Top‐down as well as cross‐section SEM analysis, including EDX was performed using a Thermo Fisher Scientific Helios 5 CX FIBSEM instrument equipped with an Oxford Instruments Ultim Max 100 mm^2^ X‐ray spectrometer. The measurements were performed on not coated sample mounted using a clamping holder. In order to prepare the cross‐section Focused Ion Beam (FIB) milling was performed. First, a carbon protection cap was deposited using 30 kV, 0.23 nA Ga ion beam. Then the cross section was opened with a 2.5 nA beam current and subsequently polished with 0.79 nA.

### 4.13 Current–Voltage Measurements

The *I*–*V* curves of devices were collected with Keysight 2902B SMU using a homemade photodetector testing setup with a C‐10 W RGB‐C series (LCFOCUS) LED as a light source using the red (620 nm) channel (Figure S5). The light flux was calibrated by measuring photocurrents with the FDS1010 photodiode from Thorlabs with a known responsivity spectrum. All *I*–*V* measurements were done in a nitrogen‐filled glovebox. The *I*–*V* sweeps were performed at 200 mV s^−1^ rates first under dark and then under illuminated (1 mW cm^−2^) conditions.

### 4.14 Linearity and Detectivity Measurements

For linearity measurements, the photodetectors were illuminated with pulsed light generated by a 461 nm 10 W RGB LED operated at 80 Hz with a 50% duty cycle. The incident optical power was varied by adjusting the LED drive current and using calibrated neutral‐density filters. The optical power per pulse was calibrated using a reference silicon photodiode (FDS1010 photodiode from Thorlabs).

The resulting photocurrent was amplified using a FEMTO DLPCA‐200 transimpedance amplifier with gains of up to 10^11^ V A^−1^. Time‐domain current traces were recorded using a PicoScope 2208B with a total acquisition time of 1 s and 20 000 data points.

For detectivity measurements, the noise‐equivalent power (NEP) was calculated from the current noise spectral density obtained by Fourier transforming the recorded time‐domain traces. The effective noise bandwidth was defined as the frequency range from 10 to 100 Hz. To validate the noise analysis procedure, reference measurements were performed using precision resistors, confirming the expected Johnson–Nyquist noise levels within the same measurement bandwidth.

### 4.15 Image Sensor Testing

For cross‐talk measurements, a reference unpatterned image sensor was used with an identical procedure as for the patterned sensor. The total sensor active area was 10 × 10 mm^2^, consisting of 400 × 400 pixels. The cross‐talk tests were performed using a basic slanted‐edge technique [38]. Briefly, a diagonal bar image was positioned in front of the sensor and focused on using a lens, after that, the cross‐talk was calculated from the resulting images. To calculate the sensitivity, dynamic range, and linearity, the sensor was exposed to a uniform blue light of 460 nm with a known irradiance, and 50 images were collected for every irradiance value, starting from a minimal undetectable light intensity to a full well saturation. All photographs were acquired in an open laboratory environment using a DSL318B‐650‐F2.4 lens.

### 4.16 Atomic‐Force Microscopy

Atomic Force Microscopy (AFM) imaging was performed using an NX‐10 Park AFM with AC160TS tips in non‐contact mode, at a 0.5 Hz scan rate over a 2 × 2 µm area, capturing 256 points per row.

### 4.17 Statistical Analysis

Most measurements in this work represent device‐level characterization of representative samples, and therefore, no hypothesis‐driven statistical testing was applied unless otherwise stated. Device performance parameters were typically extracted from individual devices unless explicitly indicated.

For datasets where multiple samples were evaluated, _data_ are presented as mean ± standard deviation (SD). The number of independent samples (n) corresponds to the number of independently fabricated devices or measurements and is specified in the corresponding figure captions or supplementary figures.

No data transformations or normalization procedures were applied unless explicitly stated in the text. Outliers were not excluded from the analysis.

## Author Contributions

S.T. developed the concept of this study; S.T, X.L, T.S., and E.W. performed fabrication of samples and characterization; E.W performed SEM and CMP of ROICs; Q.N.D.L. performed lithography on the CMOS chip and assisted with sensor tests; B.T measured XRD, G.M measured detector speed, S.F and S.S measured XPS, K.C and T.J designed the CMOS chip and PCB boards, developed the image capturing software, and supported sensor testing; E.H. developed scripts for CMOS chip testing; S.T., S.Y., and M.K. wrote the manuscript with the contribution of all co‐authors. M.K., S.Y., I.S., and T.J. supervised the work. All authors discussed the results and commented on the manuscript.

## Conflicts of Interest

The authors declare no conflict of interest.

## Supporting information




**Supporting File**: adma72803‐sup‐0001‐SuppMat.docx.

## Data Availability

The data that support the findings of this study are available from the corresponding author upon reasonable request. Restrictions apply to the availability of certain files (e.g., ROIC designs) due to third‐party intellectual‐property considerations.
