# Supplementary material for: Patterning of Lead Halide Perovskite Device Stacks on CMOS Readout Using Selective Microfabrication Protocols
Source: Adv Mater. 2026 Mar 18;38(21):e23002. doi: 10.1002/adma.202523002 (PMC13073071; doi:10.1002/adma.202523002)
Supplement: Supplementary file 1 — Supporting File: adma72803‐sup‐0001‐SuppMat.docx. [file ADMA-38-e23002-s001.docx]

**Supplementary information for**

**Patterning of Lead Halide Perovskite Device Stacks on CMOS Readout Using Selective Microfabrication Protocols**

Sergey Tsarev^1,2^, Erfu Wu^1,3^, Kyuik Cho^4^, Xuqi Liu^1^, Quang Nhat Dang Lung^1^, Emeric Hartman^1^, Tian Sun^1^, Bekir Turedi^1,2^, Gebhard Matt^1,2^, Stefanie Frick^5^, Sebastian Siol^5^, Taekwang Jang^4^, Ivan Shorubalko^3^, Sergii Yakunin^1,2^, Maksym V. Kovalenko^1,2^

*^1^ Laboratory of Inorganic Chemistry, Department of Chemistry and Applied Biosciences, ETH Zürich, CH-8093 Zürich, Switzerland*

*^2^ Laboratory for Thin Films and Photovoltaics, Empa – Swiss Federal Laboratories for Materials Science and Technology, CH-8600 Dübendorf, Switzerland*

*^3^ Transport at Nanoscale Interfaces Laboratory, Empa – Swiss Federal Laboratories for Materials Science and Technology, CH-8600 Dübendorf, Switzerland*

*^4^ Laboratory of Integrated Systems, Department of Information Technology and Electrical Engineering, ETH Zürich, CH-8092 Zürich, Switzerland*

*5 Laboratory for Surface Science and Coating Technologies, Empa – Swiss Federal Laboratories for Materials Science and Technology, CH-8600 Dübendorf, Switzerland*

*E-mail: [mvkovalenko@ethz.ch](mailto:mvkovalenko@ethz.ch), [yakunins@ethz.ch](mailto:yakunins@ethz.ch)

Contents

[Supplementary Figure 1. 3](#_Toc220062856)

[Supplementary Figure 2. 3](#_Toc220062857)

[Supplementary Figure 3. 4](#_Toc220062858)

[Supplementary Figure 4. 4](#_Toc220062859)

[Supplementary Note 1. 5](#_Toc220062860)

[Supplementary Table 1. 7](#_Toc220062861)

[Supplementary Figure 5. 8](#_Toc220062862)

[Supplementary Figure 6. 9](#_Toc220062863)

[Supplementary Figure 7. 10](#_Toc220062864)

[Supplementary Figure 8. 11](#_Toc220062865)

[Supplementary Table 2. 12](#_Toc220062866)

[Supplementary Figure 9. 13](#_Toc220062867)

[Supplementary Figure 10. 14](#_Toc220062868)

[Supplementary Figure 11. 14](#_Toc220062869)

[Supplementary Figure 12. 15](#_Toc220062870)

[Supplementary Figure 13. 16](#_Toc220062871)

[Supplementary Figure 14. 16](#_Toc220062872)

[Supplementary Figure 15. 17](#_Toc220062873)

[Supplementary Figure 16. 18](#_Toc220062874)

[References 19](#_Toc220062875)


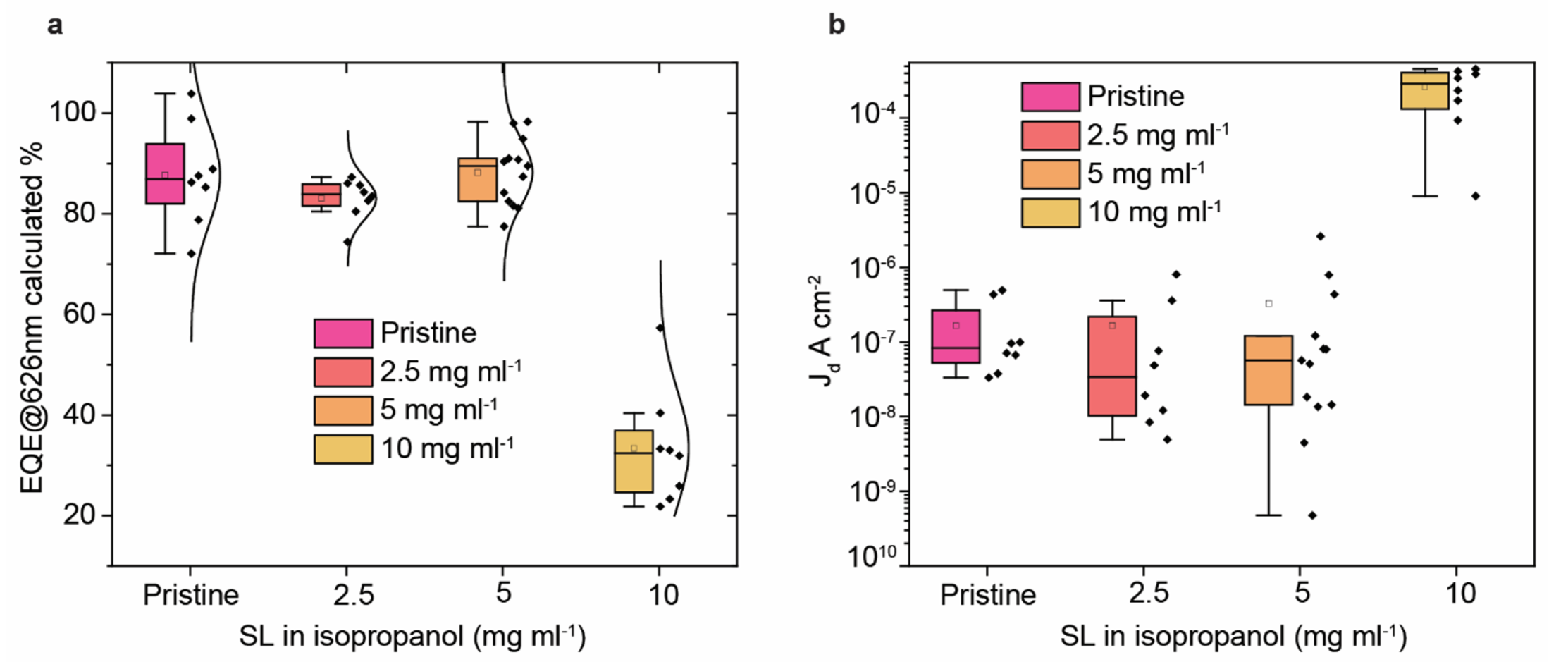


Supplementary Figure 1. External quantum efficiency (a) at 626 nm calculated from photocurrents and dark current density (b) at -500 mV reverse bias calculated from IV measurements of the devices passivated with 0-10mg ml^-1^ SL in isopropanol. Box plots represent the median (center line), interquartile range (box), and whiskers extending to the most extreme data points within 1.5× the interquartile range. Black dots correspond to individual device measurements (n = 12 devices per condition, fabricated on two substrates with six devices each)


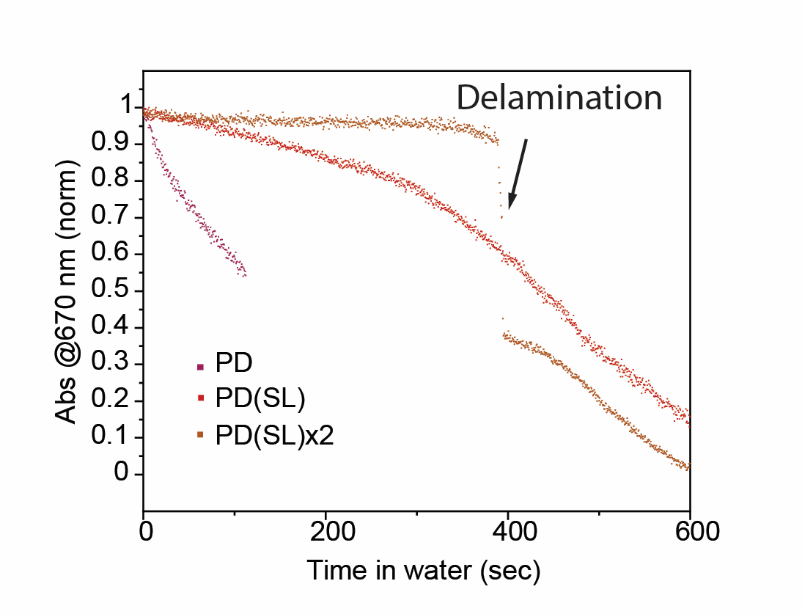


Supplementary Figure 2. Six-minute water immersion tests of device stacks with sorbitan laurate (SL) coatings applied on perovskite and additionally on top of ITO layer (SLx2). For SLx2 device, delamination eventually occurred, originating from the substrate edges.


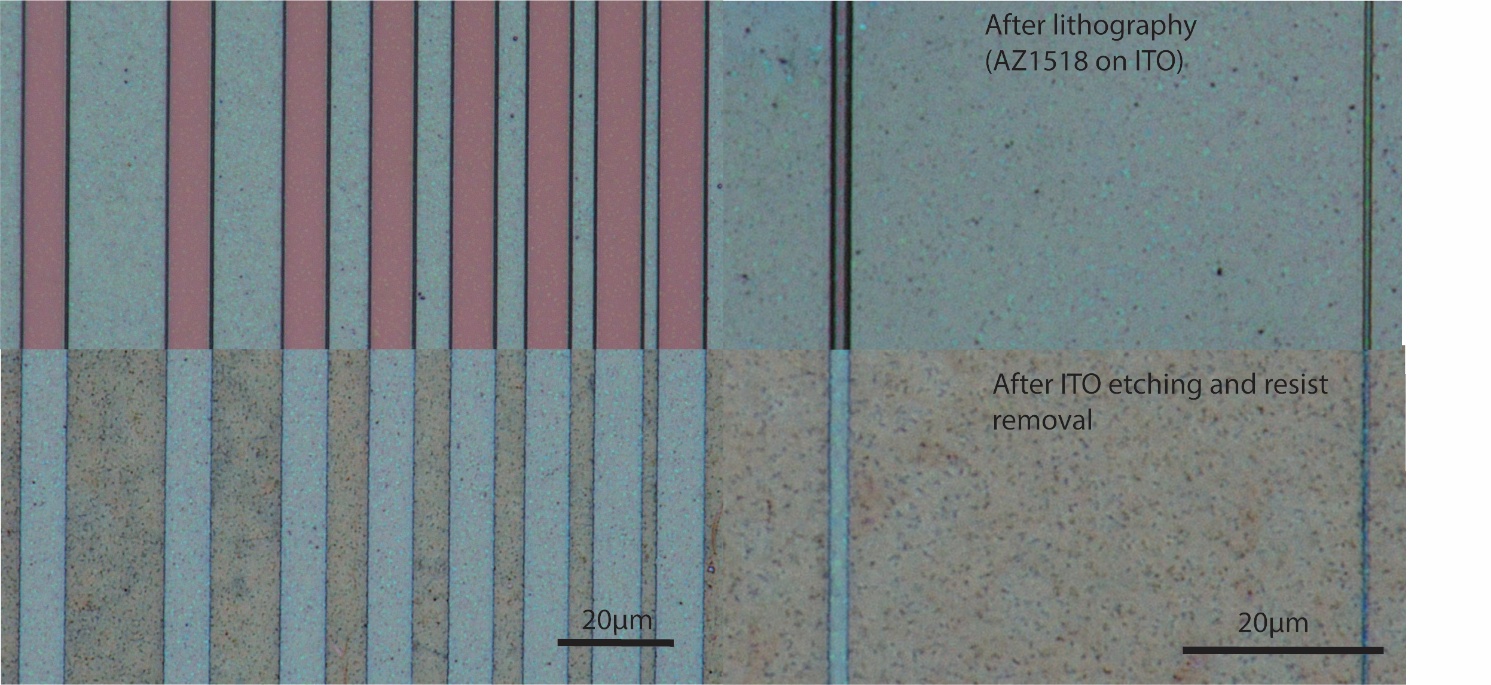


Supplementary Figure 3. AZ1505 photoresist patterns after lithography on the perovskite device stack and after etching of ITO


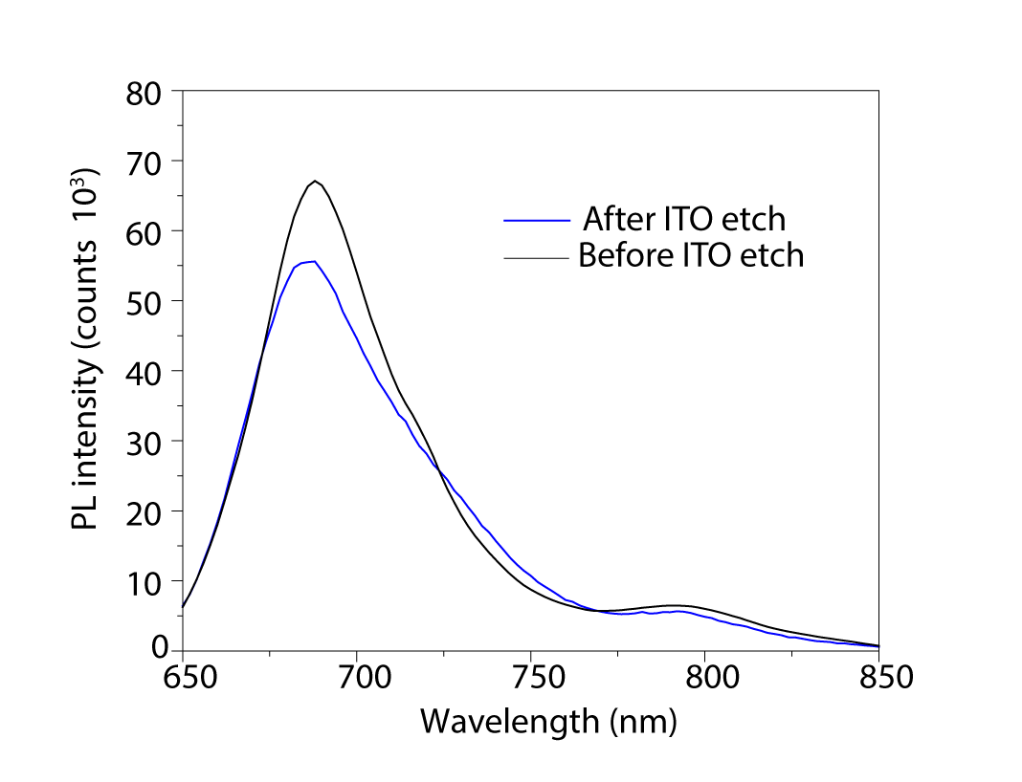


Supplementary Figure 4. PL spectra of perovskite device stack with ITO/MoO_3_/2PACz/MAPbI_2_Br/C_60_/BCP/ITO before and after the ITO etching

Supplementary Note 1. Perovskite patterning strategies for image sensors and photodetectors.

While a wide range of perovskite patterning techniques has been reported in the literature, many of these approaches face limitations when evaluated from the perspective of scalable optoelectronic device fabrication. In image sensors and photodetectors, patterning performance is not solely determined by the minimum achievable feature size, but rather by a combination of pixel pitch, edge damage, fill factor, reproducibility, and compatibility with large-area processing.

For image sensor applications, the primary objective of patterning is to electrically and optically isolate adjacent pixels while preserving the maximum active area for light absorption. Consequently, the ability to densely pack pixels with minimal dead area and minimal edge degradation is often more relevant than achieving isolated sub-micron features. Similar considerations apply to light-emitting devices, where pixel area directly impacts current density and operational stability.

In this context, several reported below (Supplementary Table 1) patterning approaches—such as surface wetting/non-wetting methods, lift-off processes, peel-off techniques, and focused ion beam (FIB) patterning—are inherently limited in scalability or packing density and are therefore not suitable for wafer-level manufacturing. When scalability and dense pixel integration are required, the set of viable approaches narrows primarily to dry etching and ion-exchange–based methods.

Dry etching techniques, including Ar milling and inductively coupled plasma (ICP) etching, are anisotropic and, in principle, offer resolution limited by lithography. However, these processes physically remove material and often introduce substantial edge damage in perovskite films. Reported SEM images ^[1]^ reveal damaged regions extending on the order of several hundred nanometers from the etched edge, rendering part of the perovskite near the pixel boundary non-photoactive. In addition, dry etching can damage underlying layers and CMOS passivation stacks (e.g., SiO_2_/SiN), and may introduce secondary sputtering artifacts, particularly problematic for monolithic integration on CMOS readout circuits. Furthermore, physical removal of lead-containing perovskite material during plasma etching raises contamination concerns, as Pb species can redeposit on chamber walls and components. Mitigating such contamination typically requires dedicated or frequently cleaned plasma tools, which are often unavailable in shared academic cleanroom environments and can significantly increase process cost and complexity. Moreover, etched perovskite edges are directly exposed to ambient conditions, which can accelerate defect formation and long-term degradation.

Ion-exchange–based patterning methods, including solution-based halide exchange and SF_6_ plasma–assisted gaseous exchange, rely on isotropic chemical conversion of the perovskite. In such processes, the lateral resolution is fundamentally limited by the perovskite film thickness. For the ~500 nm thick films used in this work, this defines a theoretical resolution limit of approximately 500 nm. In practice, plasma non-uniformity and large-area processing led to lateral overconversion exceeding 1 µm. For reliable pixel isolation across large device areas, we therefore employ pixel dimensions ≥10–15 µm, ensuring complete conversion and high yield.

Despite this resolution limitation, gaseous ion-exchange–based patterning offers several key advantages for optoelectronic devices: preservation of surface topography, absence of physically exposed perovskite edges, high selectivity, and excellent scalability to large areas. These features are particularly advantageous for perovskite CMOS-integrated image sensors, where subsequent lithography, encapsulation, and packaging steps are required.

Supplementary Table 1. Summary of perovskite patterning methods for application in CMOS image sensors.

| Perovskite patterning method | Edge damage for 500 nm film | Process type | Scalability | Selectivity | Topography preservation | References |
| --- | --- | --- | --- | --- | --- | --- |
| SF_6_ plasma halide exchange | 670 nm > | Isotropic | Yes | Yes | Yes | This work |
| Ar dry etching | Not reported | Anisotropic | Yes | No | No | ^[2]^ |
| ICP (CF_4_, Cl_2_, He) | ~ 600nm from perovskite edge | Anisotropic | Yes | Yes | No | ^[1, 3]^ |
| FIB | Not reported/ minimal | Anisotropic | No | No | Yes | ^[4]^ |
| In-situ cross linking | None | Anisotropic | Yes | Yes | No | ^[5]^ |
| Halide exchange (solution) | Not reported | Isotropic | Yes | Yes | Yes | ^[6]^ |
| Wetting/nonwetting | Not reported | N/A | No | Yes | No | ^[7]^ |
| Lift-off/Peel off | None | N/A | Yes | No | No | ^[8]^ |


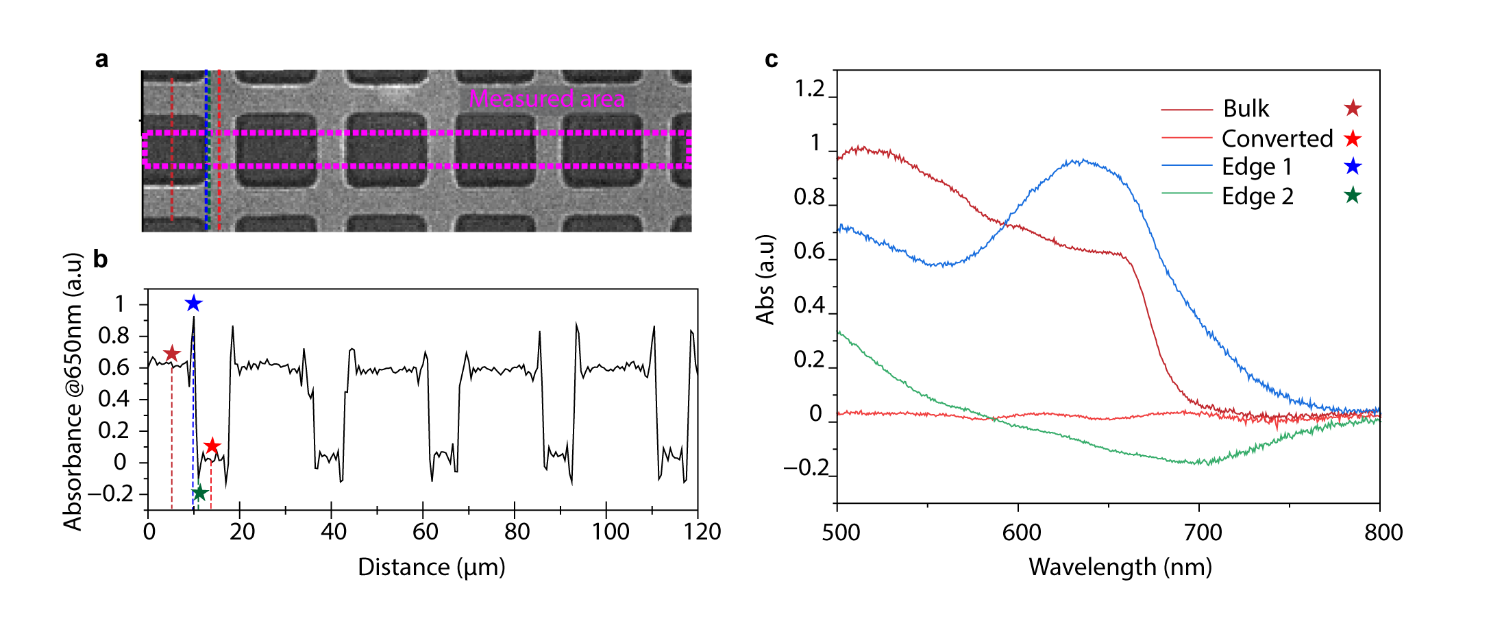


Supplementary Figure 5. Optical absorption mapping of fully converted 15 (nominal) µm perovskite square patterns (20% SF6 80% N2, 100 W, 110 sec), identical to those shown in Figure 3e. (a) Optical micrograph of the mapped area. Absorption spectra were collected along the indicated line with a lateral resolution of approximately 500 nm per pixel. (b) Lateral absorbance profile extracted at 650 nm along the scan direction. (c) Representative absorption spectra corresponding to the locations marked by stars in (b), taken from the unvonverted center of the square, the center of the fully converted region, and the vicinity of the converted edge.


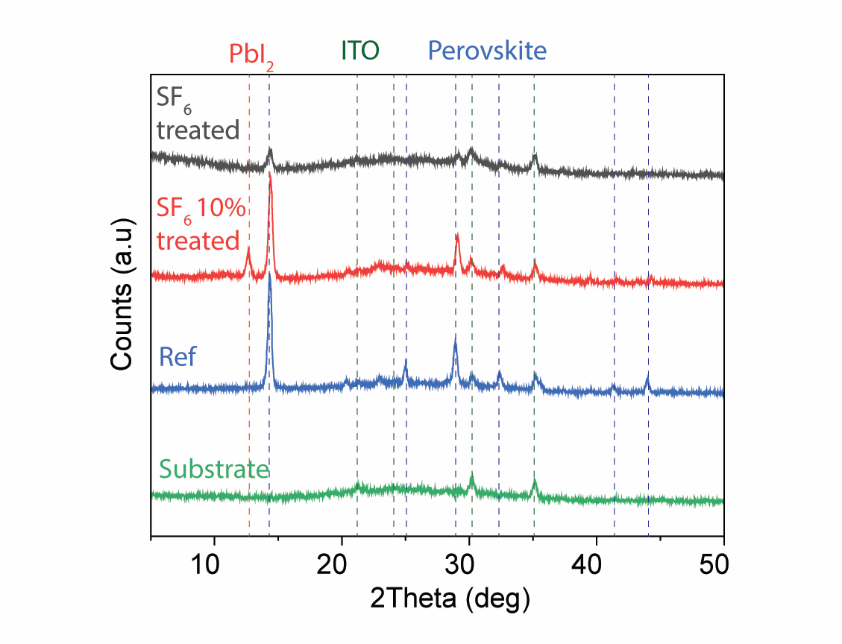


Supplementary Figure 6. X-ray diffraction patterns of ITO/MoO_3_/2PACz/MAPbI_2_Br samples after 10 seconds exposure to a. 90% N_2_ 10% SF_6_ and b. 100% SF_6_ plasma compared with an untreated perovskite and substrate (ITO) references.


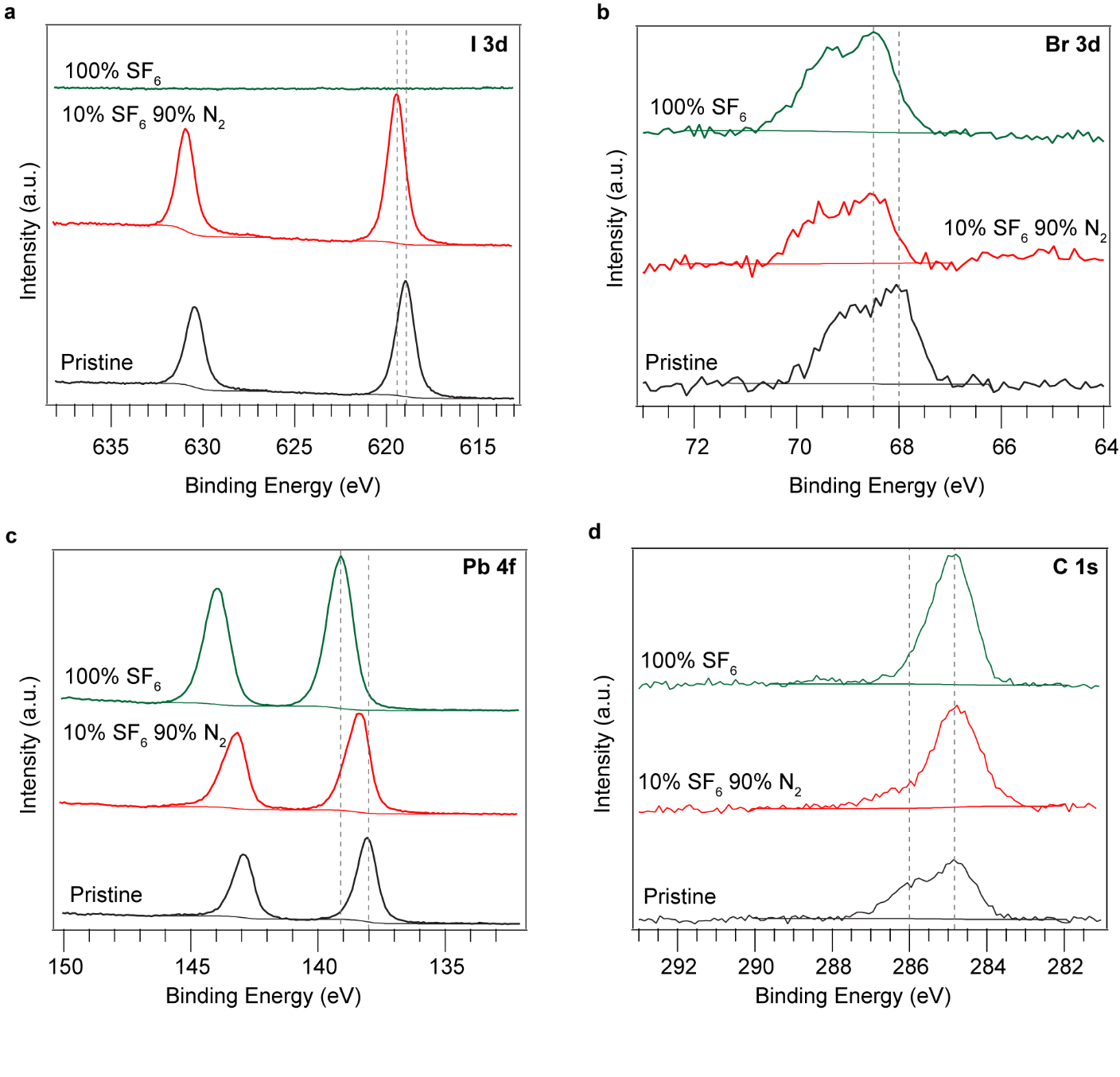


Supplementary Figure 7. XPS detail spectra of the (a) I 3d, (b) Br 3d, (c) Pb 4f, (d) C 1s regions of a pristine, a partially and a fully converted sample. All spectra have been charge referenced to the aliphatic component in the C 1s spectra at 284.8 eV ^[9]^.


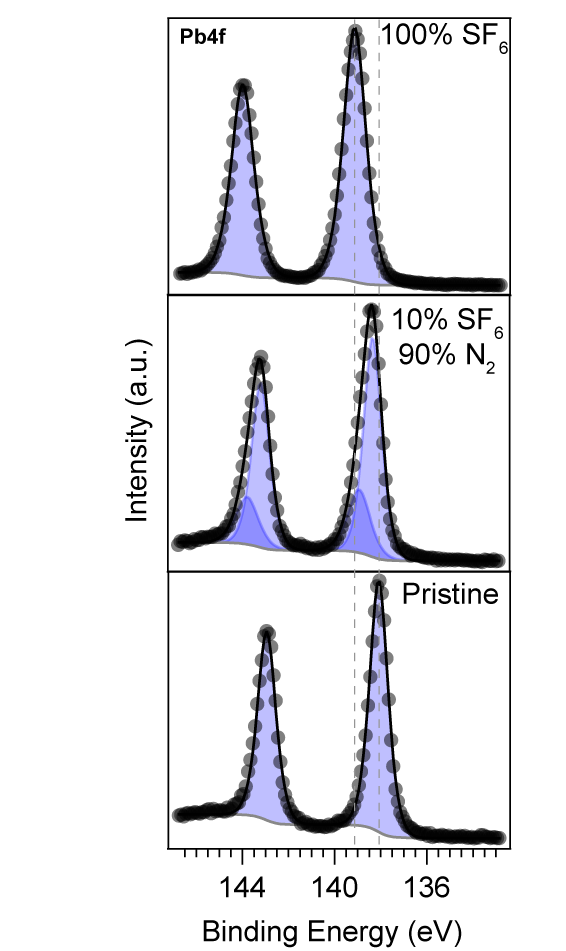


Supplementary Figure 8. Fitted Pb 4f spectra of a pristine, a partially and a fully converted sample. A Shirley back-ground and doublets with a GL(60) Voigt line shape and a spin-orbit-splitting energy of 4.86 eV [10] were applied using the Casa XPS software. Due the asymmetry of the doublet, two components were required in case of the partially converted sample. The FWHM of the two doublets were constrained to those obtained for the pristine and the fully converted sample.

Supplementary Table 2. Surface composition as obtained by XPS on each three measurement areas of a pristine, a partially and a fully converted sample. A Shirley background subtraction and relative sensitivity factors of the equipment suppliers were applied.

| X/Pb Atomic Ratios (Mean ± Std) | | | | | | | |
| --- | --- | --- | --- | --- | --- | --- | --- |
| Condition | **Statistic** | **C** | **N** | **I** | **Br** | **F** | **O** |
| Pristine | Mean | 3.42 | 0.57 | 1.76 | 0.42 | – | 0.17 |
|  | Std | 0.18 | 0.05 | 0.03 | 0.02 | – | 0.04 |
| Partially converted | Mean | 3.44 | – | 1.32 | 0.21 | 0.24 | 0.74 |
|  | Std | 0.76 | – | 0.25 | 0.04 | 0.09 | 0.23 |
| Fully converted | Mean | 2.71 | – | – | 0.23 | 1.26 | 0.32 |
|  | Std | 0.20 | – | – | 0.02 | 0.15 | 0.02 |


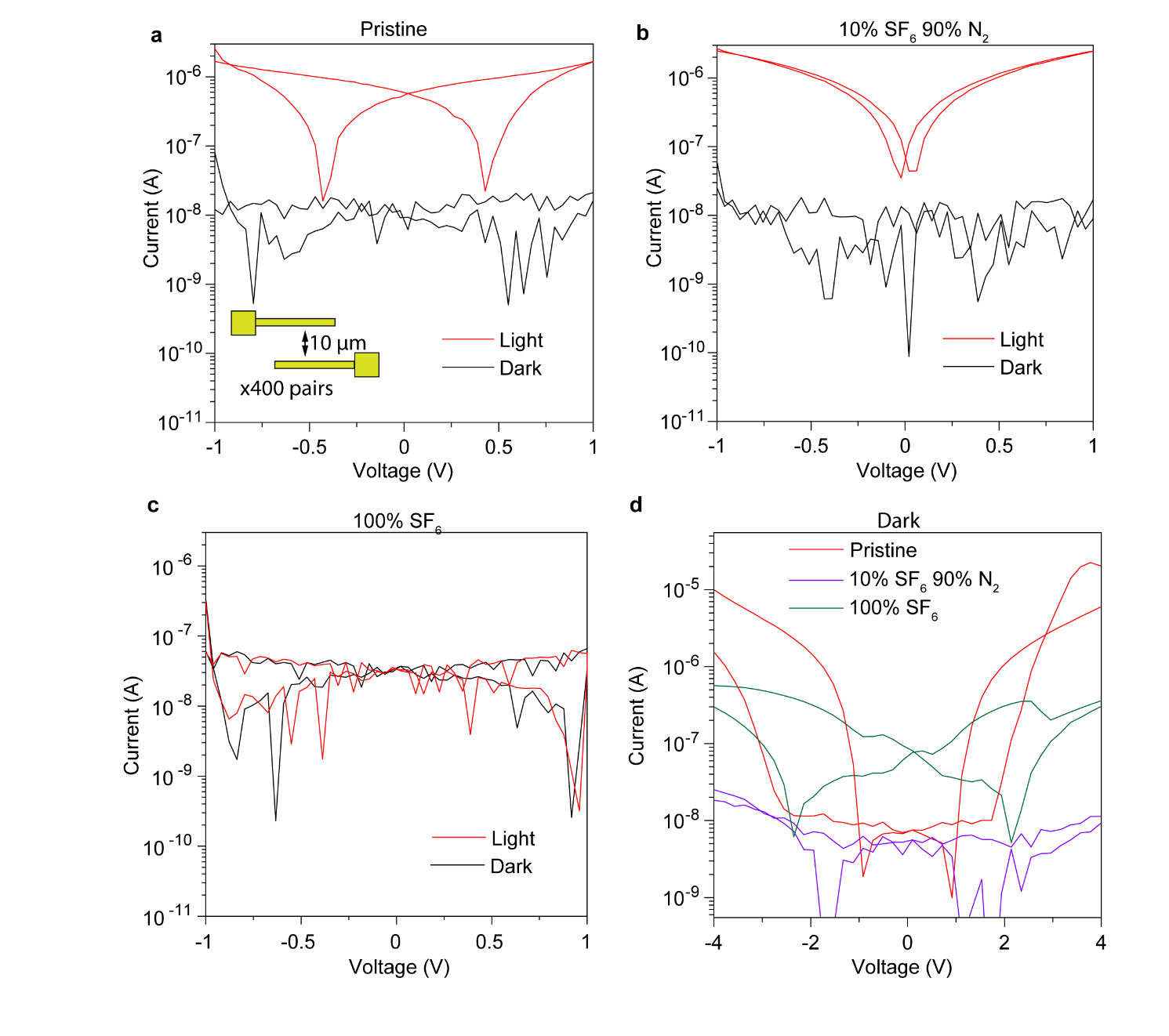


Supplementary Figure 9. Current–voltage (*I–V*) characteristics of lateral photoconductors fabricated using lithographically patterned interdigitated ITO electrodes consisting of 400 electrode pairs with a stripe length of 190 µm, stripe width of 10 µm, and an inter-electrode spacing of 10 µm. MAPbI_2_Br films were deposited onto the electrode substrates and subsequently subjected to diluted SF_6_ plasma treatment (90% N_2_ / 10% SF_6_, 100 W, 10 s) or pure SF_6_ plasma (200 W, 20 s), corresponding to pristine (a), partially converted (b), and fully converted (c) conditions, respectively. (d) Dark current measurements acquired over an extended voltage range.


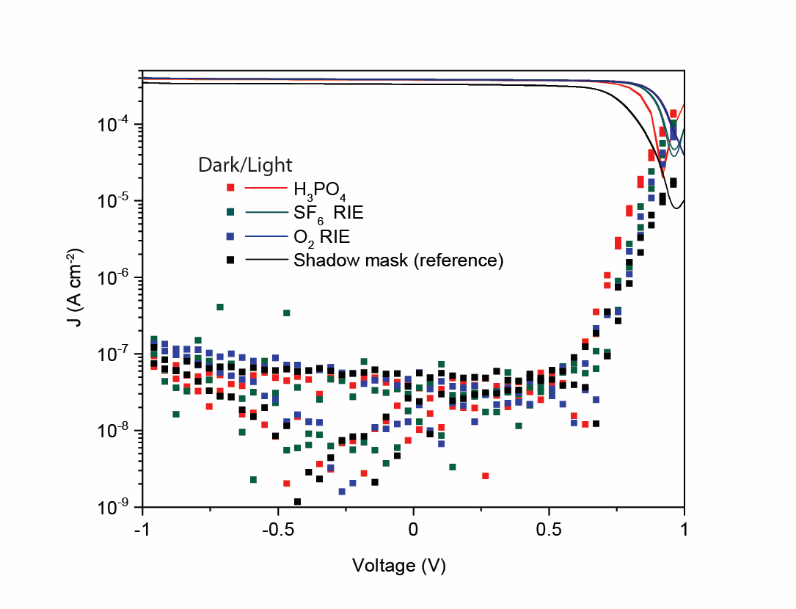


Supplementary Figure 10. Current-voltage characteristics of 4 x 4 mm^2^ lithographically patterned photodetectors *vs* a control sample, top electrode was deposited using a shadow mask.


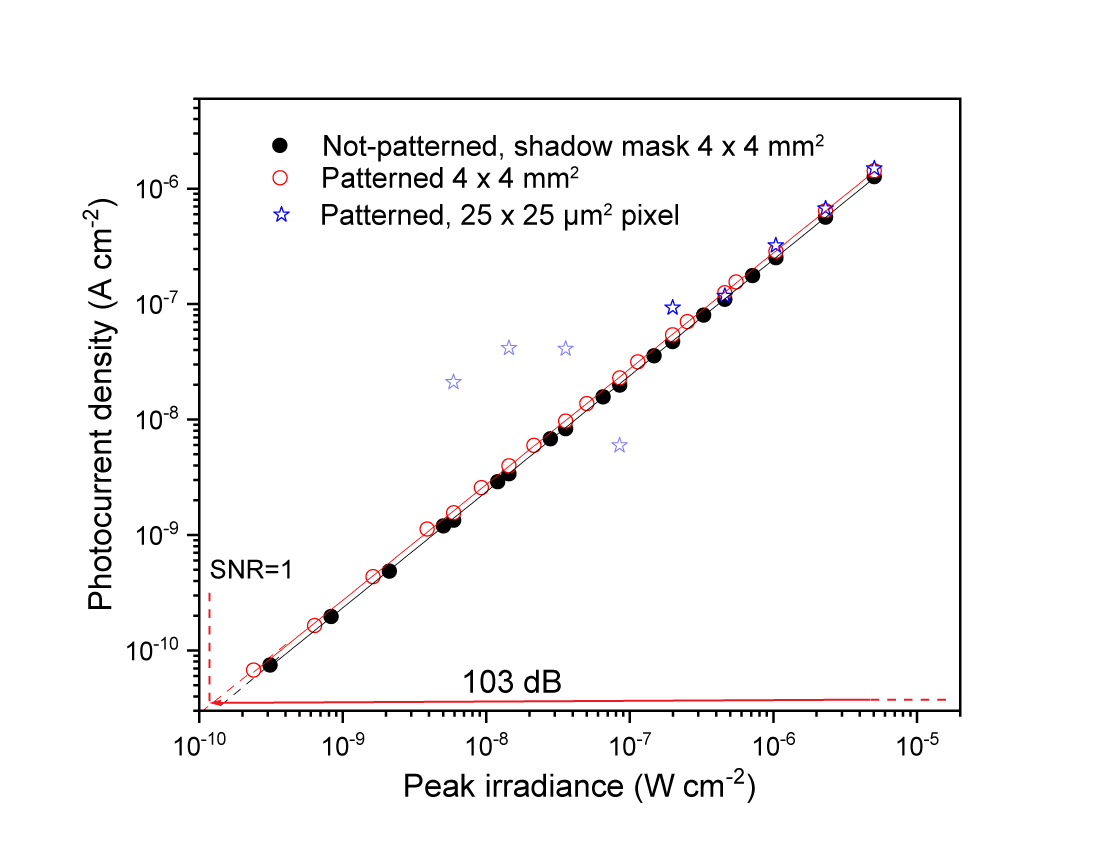


Supplementary Figure 11. Linearity measurements for 4 x 4 mm^2^ lithographically patterned photodetectors, 25 x 25 µm^2^ patterned photodetectors vs a control sample, top electrode was deposited using a shadow mask


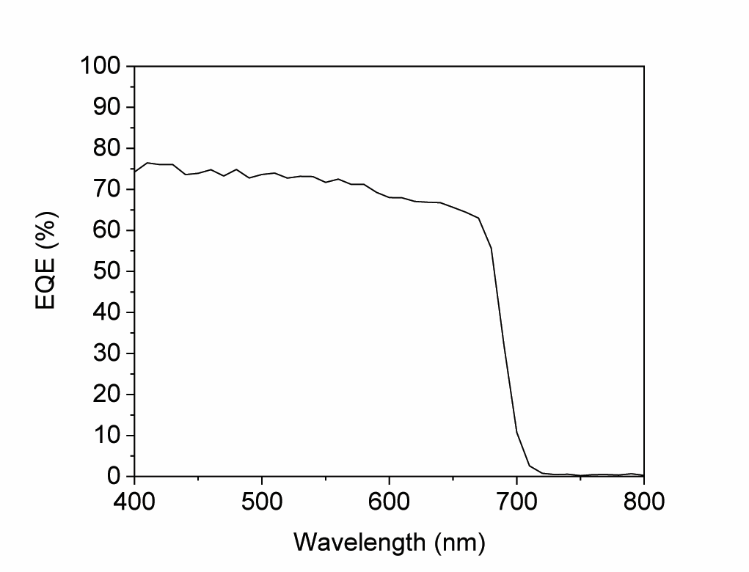


Supplementary Figure 12. External Quantum Efficiency spectrum of device with ITO/MoO_3_/2PACz/MAPbI_2_Br/C_60_/BCP/Mg/ITO/SU-8, illuminated from ITO/SU-8 side.

**Supplementary Table 3.** Comparative analysis of perovskite photodetector performance metrics.

| Figure of merit | Reported in this work | State of the art (perovskite photodiodes) |
| --- | --- | --- |
| Responsivity | 0.28 A∙W^-1^ at 460nm | 0.34 A∙W^-1^ at 461 nm ^[11]^ |
| Dark current density at 0.5 V | 80 nA∙cm^2^ | 10 pA∙cm^2 [12]^ |
| Speed (25 x 25 µm^2^), time constant | 110 ns | 0.65∙ns ^[13]^ |
| Specific Detectivity | 2.8∙10^11^ Jones | 7.5∙10^12 [14]^ at 530 nm |
| Area (patterned) | 15 x 15 µm^2^ | 1 x 1 µm^2 [15]^ |
| Linear Dynamic Range (detector) | 103∙dB | 200∙dB ^[16]^ |
| Resolution (CMOS sensor) | 400 x 400 | 768 × 512 ^[17]^ |


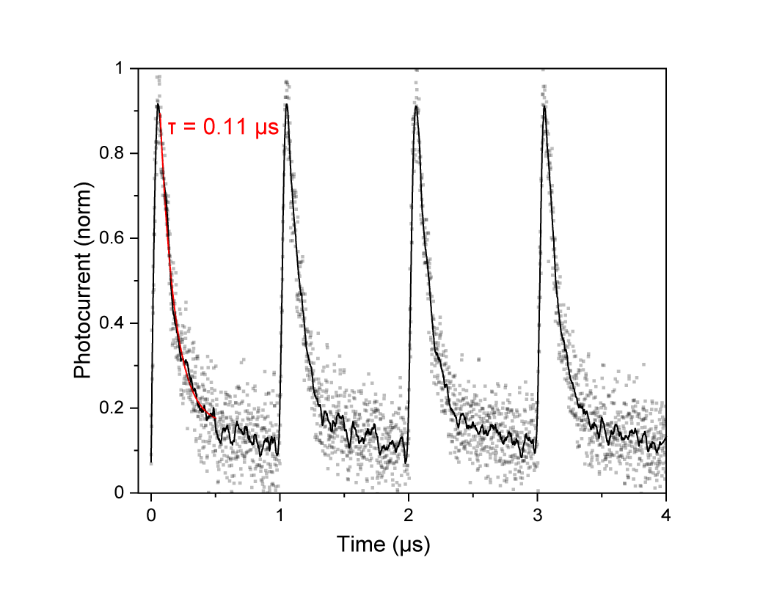


Supplementary Figure 13. Transient photocurrent response of a 25 x 25 µm^2^ lithographically patterned photodetector measured under pulsed 488 nm laser excitation.


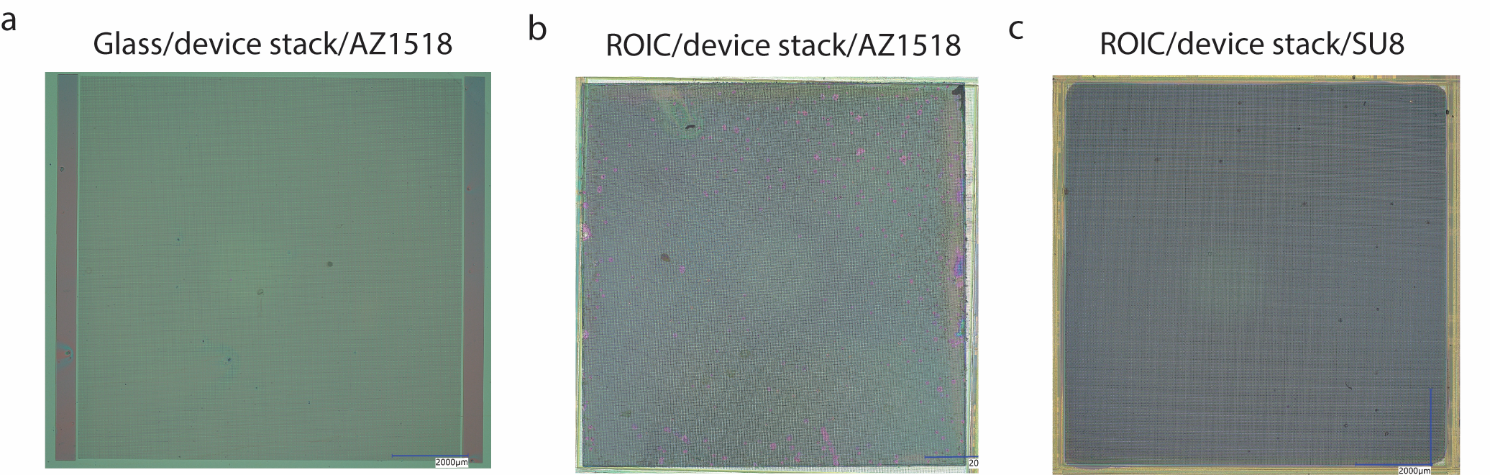


Supplementary Figure 14. Optical microphotographs of large area (11x11mm) pixel patterns on glass (a) and on CMP polished CMOS substrates coated with patterned AZ1518 (b) and SU-8 photoresists (c).


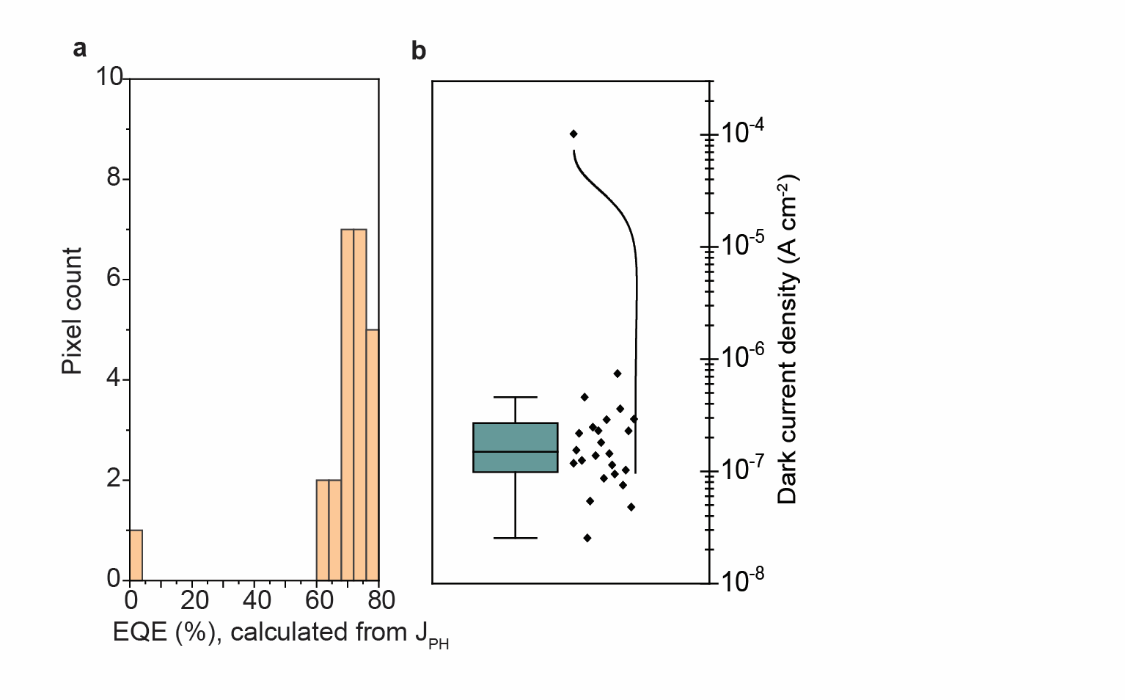


Supplementary Figure 15. Statistics of pixel yield shown as a. EQE calculated from photocurrent values, b. dark current density at -0.5 V reverse bias for MAPbI2Br detectors with ITO/MoO_3_/2PACz (evaporated)/MAPbI_2_Br/C_60_/BCP/Mg/ITO. The box plot represents the median (center line), interquartile range (box), and whiskers extending to the most extreme values within 1.5× the interquartile range. Black dots correspond to individual pixel measurements. Statistics were obtained from n = 24 devices fabricated on four independent substrates (six pixels per substrate).


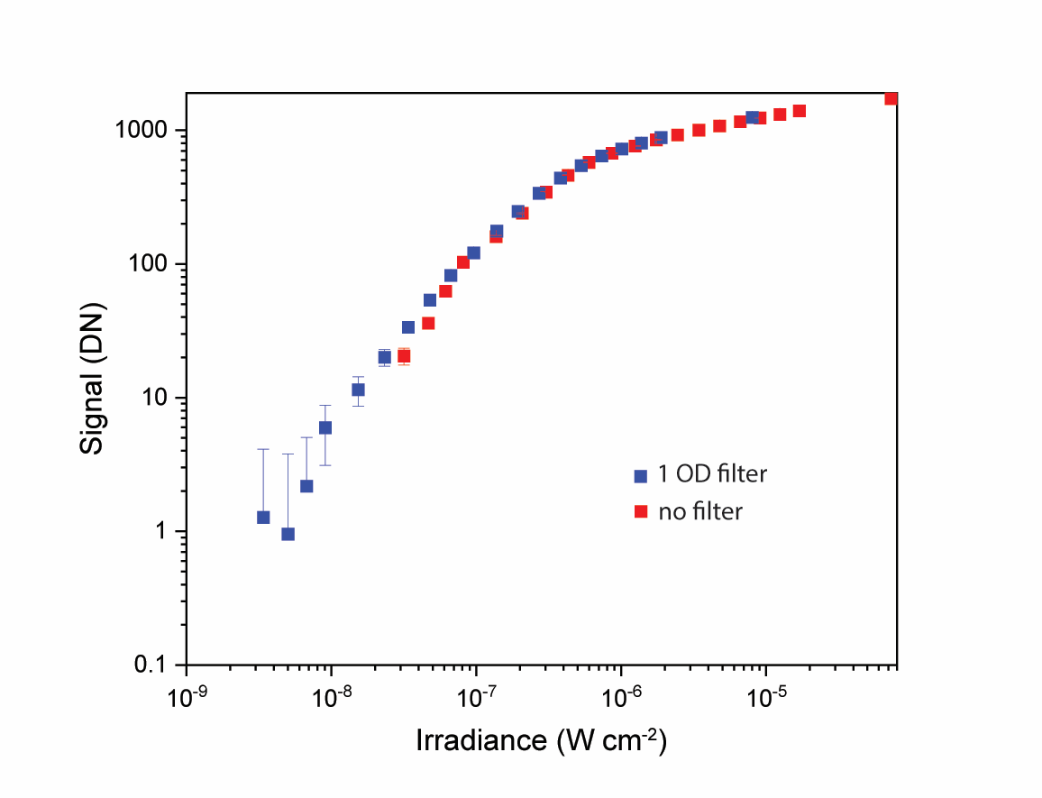


Supplementary Figure 16. Linear dynamic range of the CMOS perovskite sensor measured as signal in DN vs irradiance in W cm^-2^ (460 nm LED). For each irradiance level, 50 frames were recorded. For every pixel, the mean signal was first calculated across the recorded frames. The reported sensor signal corresponds to the median of the pixel-mean values, while the error bars represent the standard deviation of the pixel means, reflecting pixel-to-pixel response variation.

### References

[1] F. Fabrizi, S. Goudarzi, S. Khan, T. Mohammad, L. Starodubtceva, P. J. Cegielski, F. Thiel, S. Ozen, M. Schiffer, F. Lang, P. H. Bolivar, T. Riedl, G. Muller-Newen, S. B. Anantharaman, M. Mohammadi, M. C. Lemme, A Versatile Top-Down Patterning Technique for Perovskite On-Chip Integration, *ACS Nano* **2025**, 19, 30428.

[2] J. Harwell, J. Burch, A. Fikouras, M. C. Gather, A. Di Falco, I. D. W. Samuel, Patterning Multicolor Hybrid Perovskite Films via Top-Down Lithography, *ACS Nano* **2019**, 13, 3823.

[3] P. J. Cegielski, A. L. Giesecke, S. Neutzner, C. Porschatis, M. Gandini, D. Schall, C. A. R. Perini, J. Bolten, S. Suckow, S. Kataria, B. Chmielak, T. Wahlbrink, A. Petrozza, M. C. Lemme, Monolithically Integrated Perovskite Semiconductor Lasers on Silicon Photonic Chips by Scalable Top-Down Fabrication, *Nano Lett.* **2018**, 18, 6915.

[4] M. S. Alias, Y. Yang, T. K. Ng, I. Dursun, D. Shi, M. I. Saidaminov, D. Priante, O. M. Bakr, B. S. Ooi, Enhanced Etching, Surface Damage Recovery, and Submicron Patterning of Hybrid Perovskites using a Chemically Gas-Assisted Focused-Ion Beam for Subwavelength Grating Photonic Applications, *J Phys Chem Lett* **2016**, 7, 137.

[5] J. Guo, J. Wang, S. Chen, P. Tong, Y. Liu, Y. Zhang, X. Zhang, Patterning luminescent and stable perovskite-acrylic polymer composites via a convenient strategy, *APL Materials* **2024**, 12.

[6] L. Helmbrecht, M. H. Futscher, L. A. Muscarella, B. Ehrler, W. L. Noorduin, Ion Exchange Lithography: Localized Ion Exchange Reactions for Spatial Patterning of Perovskite Semiconductors and Insulators, *Adv. Mater.* **2021**, 33, e2005291.

[7] W. Lee, J. Lee, H. Yun, J. Kim, J. Park, C. Choi, D. C. Kim, H. Seo, H. Lee, J. W. Yu, W. B. Lee, D. H. Kim, High-Resolution Spin-on-Patterning of Perovskite Thin Films for a Multiplexed Image Sensor Array, *Adv. Mater.* **2017**, 29, 1702902.

[8] W. Lee, Y. J. Yoo, J. Park, J. H. Ko, Y. J. Kim, H. Yun, D. H. Kim, Y. M. Song, D. H. Kim, Perovskite microcells fabricated using swelling-induced crack propagation for colored solar windows, *Nat. Commun.* **2022**, 13, 1946, 1946.

[9] M. C. Biesinger, Accessing the robustness of adventitious carbon for charge referencing (correction) purposes in XPS analysis: Insights from a multi-user facility data review, *Appl Surf. Sci.* **2022**, 597, 153681.

[10] D. Briggs, Handbook of X-ray Photoelectron Spectroscopy C. D. Wanger, W. M. Riggs, L. E. Davis, J. F. Moulder and G. E.Muilenberg Perkin-Elmer Corp., Physical Electronics Division, Eden Prairie, Minnesota, USA, 1979. 190 pp. $195, *Surf. Interface Anal.* **1981**, 3, v.

[11] Z. Xiong, Q. Zhang, K. Cai, H. Zhou, Q. Song, Z. Han, S. Kang, Y. Li, Q. Jiang, X. Zhang, J. You, Homogenized chlorine distribution for >27% power conversion efficiency in perovskite solar cells, *Science* **2025**, 390, 638.

[12] R. Ollearo, J. Wang, M. J. Dyson, C. H. L. Weijtens, M. Fattori, B. T. van Gorkom, A. J. J. M. van Breemen, S. C. J. Meskers, R. A. J. Janssen, G. H. Gelinck, Ultralow dark current in near-infrared perovskite photodiodes by reducing charge injection and interfacial charge generation, *Nat. Commun.* **2021**, 12, 7277.

[13] L. Shen, Y. Fang, D. Wang, Y. Bai, Y. Deng, M. Wang, Y. Lu, J. Huang, A Self-Powered, Sub-nanosecond-Response Solution-Processed Hybrid Perovskite Photodetector for Time-Resolved Photoluminescence-Lifetime Detection, *Adv. Mater.* **2016**, 28, 10794.

[14] N. R. Al Amin, C.-C. Lee, Y.-C. Huang, C.-J. Shih, R. Estrada, S. Biring, M.-H. Kuo, C.-F. Li, Y.-C. Huang, S.-W. Liu, Achieving a Highly Stable Perovskite Photodetector with a Long Lifetime Fabricated via an All-Vacuum Deposition Process, *ACS Appl. Mater. Interfaces* **2023**, 15, 21284.

[15] D. Zheng, Z. Xie, W. Huang, D. Bai, J. Kim, D. Zhao, F. Qin, D. Zhang, J.-S. Kim, J. Chen, Y. Yao, Z. Wang, S. Sakshi, J.-P. Correa-Baena, L. J. Lauhon, M. G. Kanatzidis, T. J. Marks, A. Facchetti, Ultra-Flexible Pixelated Perovskite Photodetectors Enabled by Honeycomb Polymer Grids for High-Resolution Imaging, *Adv. Mater.* **2025**, 37, 2415068.

[16] P. D. Khanikar, S. Shah, A. Shukla, C. M. Cole, Z. Guo, S. Das, S.-C. Lo, T. Matsushima, E. B. Namdas, Unified Approach to Sensitive and Fast Perovskite Photodetectors Featuring an Ultrawide Linear Dynamic Range, *Adv. Opt. Mater.* **2025**, 13, e01967.

[17] W. Song, J. Kang, K. Elkhouly, S. Hamdad, X. Zhang, M. I. Pintor Monroy, A. B. Siddik, P. Carolan, S. Subramaniam, Y. Kuang, F. De Roose, E. Vandenplas, N. Chandrasekaran, J. H. Kim, R. Gehlhaar, S.-J. Kim, J. Lee, J. Genoe, Halide Perovskite Photodiode Integrated CMOS Imager, *ACS Nano* **2024**, 18, 35520.
